# Comparative genomic insights into the epidemiology and virulence of plant pathogenic pseudomonads from Turkey

**DOI:** 10.1099/mgen.0.000585

**Published:** 2021-07-06

**Authors:** Marcus M. Dillon, Tatiana Ruiz-Bedoya, Cedoljub Bundalovic-Torma, Kevin M. Guttman, Haejin Kwak, Maggie A. Middleton, Pauline W. Wang, Sumer Horuz, Yesim Aysan, David S. Guttman

**Affiliations:** ^1^​ Department of Cell and Systems Biology, University of Toronto, Toronto, Ontario, Canada; ^2^​ Centre for the Analysis of Genome Evolution and Function, University of Toronto, Toronto, Ontario, Canada; ^3^​ Department of Plant Protection, Erciyes University, Kayseri, Turkey; ^4^​ Department of Plant Protection, University of Çukurova, Adana, Turkey; ^‡^​ Present address: Department of Biology, University of Toronto at Mississauga, Mississauga, Ontario, Canada

**Keywords:** bacterial diseases, phytotoxins, plant pathogens, *Pseudomonas fluorescens*, *Pseudomonas syringae*, type III secreted effectors

## Abstract

*Pseudomonas* is a highly diverse genus that includes species that cause disease in both plants and animals. Recently, pathogenic pseudomonads from the *Pseudomonas syringae* and *Pseudomonas fluorescens* species complexes have caused significant outbreaks in several agronomically important crops in Turkey, including tomato, citrus, artichoke and melon. We characterized 169 pathogenic *Pseudomonas* strains associated with recent outbreaks in Turkey via multilocus sequence analysis and whole-genome sequencing, then used comparative and evolutionary genomics to characterize putative virulence mechanisms. Most of the isolates are closely related to other plant pathogens distributed among the primary phylogroups of *P. syringae*, although there are significant numbers of *P. fluorescens* isolates, which is a species better known as a rhizosphere-inhabiting plant-growth promoter. We found that all 39 citrus blast pathogens cluster in *P. syringae* phylogroup 2, although strains isolated from the same host do not cluster monophyletically, with lemon, mandarin orange and sweet orange isolates all being intermixed throughout the phylogroup. In contrast, 20 tomato pith pathogens are found in two independent lineages: one in the *P. syringae* secondary phylogroups, and the other from the *P. fluorescens* species complex. These divergent pith necrosis strains lack characteristic virulence factors like the canonical tripartite type III secretion system, large effector repertoires and the ability to synthesize multiple bacterial phytotoxins, suggesting they have alternative molecular mechanisms to cause disease. These findings highlight the complex nature of host specificity among plant pathogenic pseudomonads.

## Data Summary

All sequenced genomes from this study are available from GenBank through BioProject PRJNA680595 (accession numbers SAMN16885796–SAMN16885853).

**Impact Statement**

Plant pathogenic diseases often emerge without warning and can have devastating effects on global food security. However, the evolutionary origins and virulence mechanisms that drive agricultural outbreaks are usually unknown. Bacteria from the *Pseudomonas syringae* and *Pseudomonas fluorescens* species complexes include some of the most globally significant agricultural pathogens, and are driving a growing number of outbreaks on fruit and vegetable crops across Turkey. Here, we compare the genomes of bacterial strains driving several of these disease outbreaks. We show that distantly related strains can cause disease on the same crop, and that many pathogenic strains lack characteristic virulence factors like the type III secretion system and bacterial phytotoxins. Our results highlight the complex nature of host–pathogen interactions and suggest that even bacteria from the same species complex that cause disease on the same host will often do so by distinct virulence mechanisms.

## Introduction

*Pseudomonas* is a highly complex genus that includes hundreds of described species, some of which cause devastating disease in both plants and animals [1–6]. Strains from the *Pseudomonas syringae* and *Pseudomonas fluorescens* species complexes are among the most commonly found bacteria associated with plants [7, 8]. *P. syringae* is primarily known for its ability to cause a wide spectrum of diseases on many agronomically important crops [2, 6], but is also recovered from non-agricultural land and aquatic environments [3, 9–11]. However, *P. fluorescens* is more commonly known as a biocontrol [12, 13] or commensal bacterium associated with the plant rhizosphere [14, 15], although some strains are known to cause important diseases in animals (including humans), plants and fungi [16–23]. This phenotypic diversity is also reflected in their genomes, as the *P. syringae* and *P. fluorescens* species complexes are two of the most diverse *Pseudomonas* lineages [14, 15, 24–27].

The *P. syringae* species complex currently consists of at least 13 evolutionarily distinct phylogroups based on both multilocus sequence analysis (MLSA) and whole-genome data [24, 28]. Seven of these (phylogroups 1, 2, 3, 4, 5, 6 and 10) share a more recent common ancestor and have been termed primary phylogroups, as they include the majority of the recognized type and pathotype strains and most strains that infect agronomically important crops [24]. Secondary phylogroups (7, 8, 9, 11, 12 and 13) are quite divergent and as a result are frequently assigned other species names. Other distinguishing features of secondary phylogroup strains include their frequent isolation from environmental sources and their lack of some of the well-known virulence factors that are conserved in primary phylogroup pathogens [24]. The *P. fluorescens* species complex has similarly been divided into nine species groups based on molecular genetic analyses [14, 15], including: *P. fluorescens*, *Pseudomonas gessardii*, *Pseudomonas fragi*, *Pseudomonas mandelii*, *Pseudomonas jessenii*, *Pseudomonas koreensis*, *Pseudomonas corrugata*, *Pseudomonas chlororaphis* and *Pseudomonas protegens*. Within these species and/or phylogroups, plant-associated strains in both the *P. syringae* and *P. fluorescens* species complexes are also frequently assigned to pathovars based on their host of isolation and characteristic disease symptoms [29–32].

Unfortunately, the taxonomy of strains in both the *P. syringae* and *P. fluorescens* species complexes is frequently found to be inconsistent with the evolutionary (i.e. phylogenetic) relationship, particularly when names are assigned based on phenotypic characteristics such as host of isolation or disease symptoms. For example, pathogenic strains that cause pith necrosis on tomato have been identified in both the *P. fluorescens* and *P. corrugata* phylogroups [16, 17]. Similarly, among *P. syringae* pathogens, there are several cases where highly divergent strains from distinct phylogroups can infect the same host (e.g. bean halo blight pathogens), as well as cases where closely related strains belonging to the same phylogroup infect distinct hosts [1, 14, 15, 24, 33].

The disparity between genetic and phenotypic relatedness raises interesting questions about the basis of host-selectivity and the capacity for host-switching among *P. syringae* and *P. fluorescens* species. One factor enabling new outbreaks of *P. syringae* and *P. fluorescens* across a broad range of hosts may be the rapid evolutionary turnover and horizontal transfer of key virulence factors [24, 34]. Type III secreted effectors (T3SEs) are a particularly notable suite of virulence factors in *P. syringae* that are injected into the host cytoplasm by the type III secretion system (T3SS) to suppress basal immunity and facilitate pathogen growth [35–38]. However, in response, plants have evolved immune surveillance mechanisms to recognize and respond to these T3SEs with a secondary layer of immunity called effector-triggered immunity (ETI) [37, 39–41]. Consequently, T3SEs are double-edged swords for pathogens. They can both enhance bacterial virulence or elicit host immunity depending on the specific genetic makeup of both the pathogen and the host. Ultimately, this interaction plays a critical role in determining the range of hosts that any given *Pseudomonas* strain can infect [34, 42]. Pseudomonads also have a number of more general virulence factors, such as phytotoxins, that directly attack plant cells to promote pathogen fitness [43]. However, unlike T3SEs, these virulence factors generally do not elicit specific host immune responses and, therefore, may facilitate the evolution of strains with broader host ranges. The collective virulence arsenal of T3SEs and phytotoxins in any given *Pseudomonas* strain can inform our understanding of the strategy employed by that strain to manipulate and extract resources from its host.

A number of different virulence strategies are pursued within the *P. syringae* species complex. The canonical tripartite T3SS (tripartite pathogenicity island, T-PAI) is conserved among the vast majority of primary phylogroup strains, and the majority of these strains have large T3SE repertoires [24, 34]. Phylogroup 2, however, is notably different from other primary *P. syringae* phylogroups, because strains from this phylogroup tend to have comparatively smaller T3SE repertoires but synthesize a greater number of phytotoxins [1, 24, 44]. Secondary phylogroup *P. syringae* strains (phylogroups 7, 8, 9, 11, 12 and 13) typically have different versions of the T3SS and very small repertoires of both effectors and phytotoxins, suggesting distinct mechanisms of virulence [24, 28]. Whether T3SEs and phytotoxins also play a dominant virulence role in pathogens from the *P. fluorescens* species complex has received much less attention.

Here, we used comparative genomics to analyse the emergence and dissemination of plant pathogenic pseudomonads in Turkey on a wide range of agronomically important hosts. Plant pathogenic *P. syringae* strains have caused a number of devastating outbreaks on vegetable and fruit crops over the past several decades in Turkey, including tomato [45], stone fruits [46], citrus [47], bean [48], pea [49] and parsley [50]. *Pseudomonas viridiflava* [51, 52], *P. fluorescens* [17], *P. corrugata* [53] and *Pseudomonas cichorii* [54, 55] have also been isolated from infected fields and greenhouses. Additionally, the causal agent of knot disease, *Pseudomonas savastanoi*, has been reported on olive, oleander, jasmine, fontanesia, myrtle [56] and pomegranate [57] in several parts of Turkey. We used MLSA to characterize 169 isolates from 19 hosts and eight provinces in Turkey, of which a subset of 58 were whole-genome sequenced. We characterized the virulence repertoires of the sequenced strains and highlight the relative contributions of T3SEs and phytotoxins to virulence in different outbreaks. We find that the majority of pathogenic pseudomonads from Turkey are derived from primary *P. syringae* phylogroups, with the lone exceptions being pith necrosis pathogens of tomato and leaf blight pathogens of muskmelon. While many isolates that cause the same disease do cluster phylogenetically, there are also examples of convergent evolution where the same disease originates in distinct phylogroups. Given the stark differences in the T3SE and phytotoxin repertoires between strains from different phylogroups, these strains appear to be able to cause the same diseases with different molecular mechanisms.

## Methods

### Sample collection and storage

The majority of the bacteria analysed in this study were isolated from diseased plants in the following Turkish provinces from 1996 to 2018: Adana (69), Mersin (50), Hatay (38), Antalya (5), Tekirdag (3), Canakkale (1), Mugla (1) and Osmaniye (1) (Table 1 and Dataset S1, available with the online version of this article). These *Pseudomonas* samples came from multiple sources, including fields, greenhouses, nurseries, orchards and parks, and caused a number of different agronomically important diseases. Additionally, for comparative purposes, we included four *Pseudomonas* strains isolated from Germany in 1994, one sample from Holland isolated in 1990 and one sample from Switzerland isolated in 1988.

**Table 1.** Summary of the *Pseudomonas* strains isolated and analysed in this study Additional isolate metadata can be found in Dataset S1. PG, Phylogroup. NA, Not Available.

Isolate	Species/pathovar	PG	Disease	Host	Isolation province	Isolation year
YA0001	*viridiflava*	7	Pith necrosis	Tomato	Mersin	2002
YA0002	*cichorii*	11	Pith necrosis	Tomato	Mugla	2003
YA0006	*apii*	5	Bacterial leaf spot	Parsley	Hatay	2012
YA0007	*apii*	5	Bacterial leaf spot	Parsley	Mersin	2011
YA0008	*pisi*	2	Bacterial blight	Pea	Adana	2016
YA0009	*pisi*	2	Bacterial blight	Pea	Adana	2014
YA0010	*tomato*	1	Bacterial speck	Tomato	Adana	2016
YA0011	*tomato*	1	Bacterial speck	Tomato	Mersin	2015
YA0012	*tomato*	1	Bacterial speck	Tomato	Adana	2015
YA0013	*tomato*	1	Bacterial speck	Tomato	Adana	2014
YA0014	*tomato*	1	Bacterial speck	Tomato	Adana	2012
YA0015	*tomato*	1	Bacterial speck	Tomato	Adana	2013
YA0016	*tomato*	1	Bacterial speck	Tomato	Hatay	1996
YA0017	*phaseolicola*	3	Halo blight	Bean	Hatay	2016
YA0018	*phaseolicola*	3	Halo blight	Bean	Hatay	2016
YA0019	*phaseolicola*	3	Halo blight	Bean	Hatay	2016
YA0020	*phaseolicola*	3	Halo blight	Bean	Hatay	2016
YA0021	*phaseolicola*	3	Halo blight	Bean	Hatay	2016
YA0022	*phaseolicola*	3	Halo blight	Bean	Hatay	2017
YA0023	*phaseolicola*	3	Halo blight	Bean	Hatay	2017
YA0024	*syringae*	2	Bacterial blight	Artichoke	Adana	2010
YA0025	*syringae*	2	Bacterial blight	Artichoke	Adana	2010
YA0026	*syringae*	2	Leaf necrosis	Muskmelon	Adana	2017
YA0027	*syringae*	2	Leaf necrosis	Muskmelon	Adana	2017
YA0030	*syringae*	2	Citrus blast	Mandarin orange	Adana	2012
YA0031	*syringae*	2	Citrus blast	Mandarin orange	Adana	2012
YA0032	*syringae*	3	Bacterial canker	Plum	Hatay	2012
YA0033	*syringae*	3	Bacterial canker	Apricot	Hatay	2014
YA0041	*syringae*	2	Citrus blast	Mandarin orange	Hatay	2014
YA0042	*syringae*	2	Citrus blast	Mandarin orange	Hatay	2014
YA0043	*syringae*	2	Citrus blast	Mandarin orange	Hatay	2014
YA0044	*syringae*	2	Citrus blast	Mandarin orange	Hatay	2014
YA0045	*syringae*	2	Citrus blast	Mandarin orange	Hatay	2014
YA0046	*syringae*	2	Citrus blast	Sweet orange	Hatay	2014
YA0047	*syringae*	2	Citrus blast	Mandarin orange	Hatay	2014
YA0048	*syringae*	2	Citrus blast	Mandarin orange	Hatay	2014
YA0049	*syringae*	2	Citrus blast	Mandarin orange	Hatay	2014
YA0050	*syringae*	2	Bacterial canker	Plum	Hatay	2014
YA0051	*syringae*	2	Bacterial canker	Plum	Hatay	2014
YA0052	*syringae*	2	Bacterial canker	Plum	Hatay	2014
YA0053	*syringae*	2	Citrus blast	Lemon	Mersin	2014
YA0054	*syringae*	2	Citrus blast	Lemon	Mersin	2014
YA0055	*syringae*	2	Citrus blast	Lemon	Mersin	2014
YA0056	*syringae*	2	Citrus blast	Lemon	Mersin	2014
YA0057	*syringae*	2	Citrus blast	Lemon	Mersin	2014
YA0058	*syringae*	2	Citrus blast	Lemon	Mersin	2014
YA0059	*syringae*	2	Citrus blast	Mandarin orange	Adana	2014
YA0060	*syringae*	2	Citrus blast	Sweet orange	Adana	2014
YA0061	*syringae*	2	Citrus blast	Mandarin orange	Adana	2014
YA0062	*syringae*	2	Citrus blast	Sweet orange	Adana	2014
YA0063	*syringae*	2	Citrus blast	Mandarin orange	Adana	2014
YA0064	*syringae*	2	Citrus blast	Sweet orange	Hatay	2015
YA0065	*syringae*	2	Citrus blast	Mandarin orange	Hatay	2015
YA0066	*syringae*	2	Citrus blast	Mandarin orange	Hatay	2015
YA0067	*syringae*	2	Citrus blast	Mandarin orange	Hatay	2015
YA0068	*syringae*	2	Citrus blast	Lemon	Hatay	2015
YA0069	*syringae*	2	Citrus blast	Sweet orange	Hatay	2015
YA0073	*syringae*	2	Citrus blast	Lemon	Mersin	2015
YA0074	*syringae*	2	Citrus blast	Lemon	Mersin	2015
YA0075	*syringae*	2	Citrus blast	Lemon	Mersin	2015
YA0076	*syringae*	2	Citrus blast	Lemon	Mersin	2015
YA0077	*syringae*	2	Citrus blast	Lemon	Mersin	2015
YA0078	*syringae*	2	Citrus blast	Lemon	Mersin	2015
YA0079	*syringae*	2	Bacterial canker	Plum	Adana	2015
YA0080	*syringae*	2	Citrus blast	Sweet orange	Adana	2015
YA0081	*syringae*	2	Citrus blast	Sweet orange	Adana	2015
YA0082	*syringae*	2	Citrus blast	Sweet orange	Adana	2015
YA0083	*syringae*	2	Citrus blast	Sweet orange	Adana	2015
YA0084	*syringae*	2	Citrus blast	Mandarin orange	Adana	2015
YA0086	*syringae*	3	Bacterial canker	Plum	Hatay	2012
YA0087	*viridiflava*	7	Pith necrosis	Tomato	Mersin	2002
YA0088	*viridiflava*	7	Leaf blight	Muskmelon	Adana	2002
YA0089	*viridiflava*	7	Leaf blight	Muskmelon	Adana	2003
YA0092	*tomato*	1	na	na	na	2014
YA0093	*tomato*	1	Bacterial speck	Tomato	Adana	2017
YA0094	*tomato*	1	Bacterial speck	Tomato	Mersin	2016
YA0186	*cichorii*	11	na	Lettuce	na	1990
YA0187	*viridiflava*	7	na	Bean	na	1988
YA0247	*savastanoi*	3	Knot disease	Oleander	Adana	2017
YA0265	*savastanoi*	3	Knot disease	Oleander	Mersin	2017
YA0278	*savastanoi*	3	Knot disease	Oleander	Mersin	2017
YA0289	*savastanoi*	3	Knot disease	Oleander	Adana	2017
YA0300	*savastanoi*	3	Knot disease	Oleander	Mersin	2017
YA0301	*savastanoi*	3	Knot disease	Oleander	Adana	2017
YA0306	*savastanoi*	3	Knot disease	Oleander	Adana	2017
YA0329	*savastanoi*	3	Knot disease	Oleander	Mersin	2017
YA0344	*savastanoi*	3	Knot disease	Oleander	Mersin	2017
YA0348	*savastanoi*	3	Knot disease	Oleander	Adana	2017
YA0359	*savastanoi*	3	Knot disease	Oleander	Adana	2017
YA0365	*savastanoi*	3	Knot disease	Oleander	Mersin	2017
YA0372	*savastanoi*	3	Knot disease	Oleander	Mersin	2017
YA0385	*savastanoi*	3	Knot disease	Oleander	Osmaniye	2017
YA0409	*savastanoi*	3	Knot disease	Olive	Mersin	2017
YA0423	*savastanoi*	3	Knot disease	Oleander	Mersin	2017
YA0450	*savastanoi*	3	Knot disease	Olive	Hatay	2017
YA0473	*savastanoi*	3	Knot disease	Fontanesia	Adana	2017
YA0479	*savastanoi*	3	Knot disease	Myrtle	Adana	2017
YA0513	*savastanoi*	3	Knot disease	Oleander	Adana	2017
YA0518	*savastanoi*	3	Knot disease	Myrtle	Adana	2017
YA0533	*savastanoi*	3	Knot disease	Oleander	Hatay	2017
YA0541	*savastanoi*	3	Knot disease	Oleander	Mersin	2017
YA0556	*savastanoi*	3	Knot disease	Olive	Canakkale	2008
YA0557	*savastanoi*	3	Knot disease	Pomegranate	Hatay	2014
YA0559	*savastanoi*	3	Knot disease	Oleander	Tekirdag	2017
YA0560	*savastanoi*	3	Knot disease	Oleander	Tekirdag	2017
YA0561	*savastanoi*	3	Knot disease	Oleander	Tekirdag	2017
YA0574	*tomato*	1	Bacterial speck	Tomato	Adana	2014
YA0575	*tomato*	1	Bacterial speck	Tomato	Adana	2014
YA0576	*tomato*	1	Bacterial speck	Tomato	Adana	2014
YA0577	*tomato*	1	Bacterial speck	Tomato	Adana	2014
YA0578	*tomato*	1	Bacterial speck	Tomato	Adana	2014
YA0579	*tomato*	1	Bacterial speck	Tomato	Adana	2014
YA0580	*tomato*	1	Bacterial speck	Tomato	Adana	2014
YA0581	*tomato*	1	Bacterial speck	Tomato	Adana	2014
YA0582	*tomato*	1	Bacterial speck	Tomato	Adana	2014
YA0583	*tomato*	1	Bacterial speck	Tomato	Adana	2014
YA0584	*tomato*	1	Bacterial speck	Tomato	Mersin	2015
YA0585	*tomato*	1	Bacterial speck	Tomato	Mersin	2015
YA0586	*tomato*	1	Bacterial speck	Tomato	Mersin	2015
YA0587	*tomato*	1	Bacterial speck	Tomato	Mersin	2015
YA0588	*tomato*	1	Bacterial speck	Tomato	Mersin	2015
YA0589	*tomato*	1	Bacterial speck	Tomato	Mersin	2015
YA0590	*tomato*	1	Bacterial speck	Tomato	Mersin	2015
YA0591	*tomato*	1	Bacterial speck	Tomato	Mersin	2016
YA0592	*tomato*	1	Bacterial speck	Tomato	Mersin	2016
YA0593	*tomato*	1	Bacterial speck	Tomato	Mersin	2016
YA0595	*tomato*	1	Bacterial speck	Tomato	Mersin	2016
YA0596	*tomato*	1	Bacterial speck	Tomato	Adana	2016
YA0597	*tomato*	1	Bacterial speck	Tomato	Adana	2016
YA0598	*tomato*	1	Bacterial speck	Tomato	Adana	2016
YA0599	*tomato*	2	Bacterial speck	Tomato	Adana	2016
YA0600	*tomato*	1	Bacterial speck	Tomato	Adana	2016
YA0601	*tomato*	1	Bacterial speck	Tomato	Adana	na
YA0602	*tomato*	1	Bacterial speck	Tomato	Antalya	2017
YA0637	*tomato*	2	Bacterial speck	Tomato	Adana	2017
YA0649	*viridiflava*	7	Pith necrosis	Bean	na	1994
YA0692	*syringae*	3	Bacterial canker	Apricot	Hatay	2014
YA0693	*syringae*	3	Bacterial canker	Apricot	Hatay	2014
YA0694	*syringae*	3	Bacterial canker	Plum	Hatay	2012
YA0695	*cichorii*	11	Pith necrosis	Tomato	Antalya	2003
YA0697	*viridiflava*	7	Pith necrosis	Tomato	Hatay	2002
YA0698	*viridiflava*	7	Leaf blight	Muskmelon	Adana	2002
YA0699	*viridiflava*	7	Leaf blight	Muskmelon	Adana	2002
YA0700	*viridiflava*	7	Leaf blight	Muskmelon	Adana	2002
YA0701	*viridiflava*	7	Leaf blight	Muskmelon	Adana	2002
YA0719	*fluorescens*	Pfl	Pith necrosis	Tomato	Mersin	2018
YA0720	*fluorescens*	Pfl	Pith necrosis	Tomato	Mersin	2018
YA0721	*fluorescens*	Pfl	Pith necrosis	Tomato	Mersin	2018
YA0729	*fluorescens*	Pfl	Pith necrosis	Tomato	Mersin	2018
YA0743	*atrofaciens*	2	Wheat rot	Wheat	na	1994
YA0745	*fluorescens*	Pfl	Pith necrosis	Tomato	na	1994
YA0748	*savastanoi*	3	Knot disease	Fontanesia	Adana	2014
YA0750	*savastanoi*	3	Knot disease	Olive	Hatay	2014
YA0751	*savastanoi*	3	Knot disease	Oleander	Adana	2014
YA0752	*savastanoi*	3	Knot disease	Oleander	Adana	2015
YA0753	*savastanoi*	3	Knot disease	Oleander	Adana	2016
YA0757	*viridiflava*	7	Pith necrosis	Tomato	Antalya	2003
YA0758	*viridiflava*	7	Pith necrosis	Tomato	Antalya	2003
YA0759	*viridiflava*	7	Pith necrosis	Tomato	Antalya	2003
YA0783	*corrugata*	Pfl	Pith necrosis	Tomato	Mersin	2018
YA0788	*corrugata*	Pfl	Pith necrosis	Tomato	Mersin	2018
YA0796	*syringae*	2	Bacterial blight	Artichoke	Adana	2010
YA0797	*syringae*	2	Bacterial blight	Artichoke	Adana	2010
YA0831	*cichorii*	11	Varnish spot	Lettuce	na	1994
YA0848	*fluorescens*	Pfl	Pith necrosis	Tomato	Mersin	2018
YA0849	*fluorescens*	Pfl	Pith necrosis	Tomato	Mersin	2018
YA0850	*fluorescens*	Pfl	Pith necrosis	Tomato	Mersin	2018
YA0851	*fluorescens*	Pfl	Pith necrosis	Tomato	Mersin	2018
YA0852	*fluorescens*	Pfl	Pith necrosis	Tomato	Mersin	2018
YA0853	*fluorescens*	Pfl	Pith necrosis	Tomato	Mersin	2018
YA0867	*syringae*	2	Seedling blight	Watermelon	Adana	2018
YA0868	*syringae*	2	Seedling blight	Watermelon	Adana	2018
YA0869	*syringae*	2	Seedling blight	Watermelon	Adana	2018
YA0870	*syringae*	2	Seedling blight	Watermelon	Adana	2018
YA0871	*fluorescens*	Pfl	na	Lettuce	Adana	2018

To isolate dominant bacterial strains from diseased plants, infected plant tissue was disinfected with 70 % ethanol and macerated in sterile 0.85 % (w/v) NaCl saline for 20 min. A loopful of the resultant suspension was then streaked for isolation onto King’s B (KB) plates and incubated at 25 °C for 48 h. Dominant colonies were then sub-cultured on KB plates and incubated for an additional 48 h for purification. Purified colonies were then grown for 48 h in liquid KB with shaking and stored in 15 % glycerol (w/v) at −80 °C.

Purified isolates were confirmed by molecular tests and pathogenicity assays were performed on each strain’s host of isolation to verify that they were indeed the pathogens driving disease [45]. Specifically, bacterial suspensions were prepared by growth on KB plates for 48–72 h at 25 °C and adjusted to a concentration of 1×10^7^ c.f.u. ml^−1^ for inoculation [46]. Suspensions of *P. syringae* pv. *tomato*, *pisi*, *apii* and *syringae* were sprayed onto healthy leaves of their host of isolation (tomato, pea, parsley, melon, watermelon) until run off [47]. Pith necrosis strains were injected into the xylem of the healthy tomato and lettuce seedlings using a sterile syringe [48, 49]. Knot disease pathogens were inoculated directly onto wounded tissue from their corresponding host of isolation (olive, oleander, mrytus, fontenesia) [50]. Finally, all citrus blast pathogens were injected into the shoot tips of 1-year-old citrus plants using a sterile syringe. All plants were kept in a greenhouse at 21–25 °C and 60–80 % humidity to allow for symptoms to develop. Anticipated disease symptoms were observed on the hosts of isolation in all strains analysed in this study and re-isolations were performed to confirm that the inoculated strain was indeed driving disease.

### MLSA

Isolates were grown with shaking in 5 ml liquid KB at 30 °C for approximately 20 h. DNA was extracted using the Gentra Puregene yeast and bacteria kit (Qiagen). DNA concentrations were determined using a spectrophotometer and diluted to 20 ng µl^−1^ for PCR amplification. Four housekeeping genes were used for the multilocus sequence typing classification: the genes encoding glyceraldehyde-3-phosphate dehydrogenase A (*gapA*), citrate synthase (*gltA*), gyrase B (*gyrB*) and RNA polymerase σ70 factor (*rpoD*). The primers used are listed in Table S1. Primers were used for PCR only (p), sequencing only (s), or both PCR and sequencing (ps). The resulting PCR products were Sanger sequenced on an AB3730 DNA Analyzer (Applied Biosystems) or sequenced on an Illumina MiSeq with 150×2 paired-end reads as previously described [51]. Sequences were aligned and trimmed to reference sequences using CLC Genomics Workbench 6 (Qiagen).

### Whole-genome sequencing and assembly

A collection of 70 representative strains from our collection were also whole-genome sequenced at the Centre for the Analysis of Genome Evolution and Function (CAGEF) at the University of Toronto, Canada. Purified DNA was extracted from each strain using a Gentra Puregene yeast and bacteria kit (Qiagen) and each DNA sample was suspended in 1x TE buffer. All purified DNA samples were quantified with a Qubit dsDNA BR assay kit (Thermo Fisher Scientific). Sequencing libraries were then generated using the Illumina Nextera XT DNA library preparation kit following the manufacturer’s instructions with Illumina dual indexes. Sequencing was performed on an Illumina NextSeq 500 with 150 base paired-end reads.

Following sequencing, low-quality bases and adapters were trimmed using Trimmomatic v.0.38 [52] (ILLUMINACLIP – NexteraPE-PE.fa, Seed Mismatch=2, Palindromic Clip Threshold=30, Simple Clip Threshold=10; SLIDINGWINDOW – Window Size=4, Required Quality=5; MINLEN – 25) and read quality was assessed with FastQC v.0.11.5 [53]. The resultant high-quality reads were then used to assemble draft genomes for each strain with default parameters in CLC assembly cell v.4.2 from CLC Genomics Workbench 6 (Qiagen). Specifically, we used surviving paired reads from Trimmomatic as paired input, surviving unpaired forward reads from Trimmomatic as unpaired input, and 1000 bp as a minimum contig length. Raw reads were then re-mapped to the remaining contigs to calculate the read coverage and all contigs were blasted against the National Center for Biotechnology Information (NCBI) non-redundant (nr) database to identify putative contaminants. Reads that mapped to suspected contaminant contigs (those that did not have a top ten hit from the genus *Pseudomonas*) were then removed from the trimmed fastq files using in-house Python scripts and these filtered fastq files were used to re-assemble the draft genome for each strain. This contaminant filtration step improved the quality of several of our assemblies by removing short, low-coverage contigs mapping to non-*Pseudomonas* species, while also identifying a subset of strains whose genome assemblies could not be recovered due to an overload of contaminating DNA. A total of 58 representative strains survived our robust quality control and were included in the whole-genome analyses presented in this study (Dataset S2).

### Orthologue prediction

Following assembly and quality control, the 58 surviving Turkey *Pseudomonas* assemblies were annotated using Prokka v.1.12 with default settings [54]. The following 21 genome assemblies were downloaded from the NCBI and also annotated using Prokka with the same parameters to evolutionarily contextualize all of the *Pseudomonas* pathogens from this study: *P. syringae* PtoDC3000 (AE016853), *P. syringae* PsyB728a (CP000075), *P. syringae* Pph1448A (NC_005773), *P. syringae* PorI (RBOG00000000), *P. syringae* PmaES4326 (AEAK00000000), *P. syringae* PcaICMP2855 (LJPW00000000), *P. syringae* PvrICMP2848 (LJRS00000000), *P. syringae* PsyCC1417 (AVEO00000000), *P. syringae* PsyCC1557 (AVEH00000000), *P. syringae* Psy0481 (QPEA00000000), *P. syringae* PgyICMP2236 (RBRO00000000), *P. chlororaphis* O6 (NZ_CM001490), *P. corrugata* F113 (NC_016830.1), *P. fluorescens* SBW25 (NC_012660), *P. fragi* P121 (NZ_CP013861), *P. gessardii* BBc6R8 (NZ_AKXH00000000), *P. jessenii* UW4 (NC_019670), *P. koreensis* PfO1 (NC_007492), *P. mandelii* JR1 (NZ_CP005960), *P. protegens* Pf5 (NC_004129) and *Pseudomonas aeruginosa* PAO1 (NZ_CP053028). Because of the diseases that they cause, the majority of strains in our collection were expected to be from the *P. syringae* species complex, which motivated us to include a sequenced *P. syringae* strain from each of the 11 phylogroups with a representative whole-genome sequence available. Furthermore, because some *P. fluorescens* complex strains can also cause tomato pith necrosis [17], we additionally included a representative strain from each of the nine *P. fluorescens* complex phylogroups. *P. aeruginosa* PAO1 was included as an outgroup for all strains in the analysis.

Orthologue prediction and analysis for the collection of 79 genomes (58 Turkey pseudomonads, 11 representative *P. syringae* complex strains, 9 representative *P. fluorescens* complex strains and 1 *P*. aeruginosa outgroup) was conducted using pirate with default settings [55]. pirate clusters genes over a range of thresholds to identify orthologues, paralogues and putative fission/fusion events, which makes it particularly well-suited to analyse our highly diverse dataset. We used a translated core-genome alignment to explore the evolutionary relationships between the strains based on core-gene polymorphisms and the binary presence–absence matrix of all gene families to explore the relationships between strains based on their pangenome content.

### Phylogenetic analysis

We generated a number of phylogenetic trees in this study based on MLSA polymorphisms for all strains, core-genome polymorphisms for whole-genome sequenced strains, pangenome content for whole-genome sequenced strains, and polymorphisms in the core-structural genes of the T3SS for whole-genome sequenced strains. All MLSA trees were generated using a concatenated nucleotide alignment of the *gapA*, *gltA*, *gyrB* and *rpoD* loci. For representative strains from the *P. syringae* species complex, the *P. fluorescens* complex and *P. aeruginosa* PAO1, MLSA regions were extracted from the whole-genome sequences using blast+ v0.2.6.0 [56, 57]. Multiple alignments for each locus were generated using muscle v.3.8.31 with default settings [58], and the resultant alignments were concatenated using an in-house Python script. Maximum-likelihood trees were then generated with FastTree v.2.1.10 [59]. Core-genome trees were generated for our collection of whole-genome sequenced strains by partitioning the core-genome alignment output from pirate to include the correct collection of strains desired for each tree. Partitioned core-genome alignments were then translated to amino acid sequences and phylogenetically informative sites were extracted using Gblocks [60]. As was the case for our MLSA trees, maximum-likelihood trees were then generated using only phylogenetically informative sites with FastTree v.2.1.10 [59]. Our pangenome tree was generated from the binary presence–absence information of all orthologue families characterized by pirate with the output binary presence–absence fasta file used as input for FastTree v.2.1.10 [59]. Finally, our T3SS tree was generated using a concatenated alignment of the following ten core T3SS structural genes for each T3SS: *hrcC*, *hrcJ*, *hrcN*, *hrcQ*, *hrcR*, *hrcS*, *hrcT*, *hrcU*, *hrcV* and *hrpV*. Independent alignments for each family were generated using muscle v.3.8.31 and the resultant alignments were concatenated using our in-house Python script. The final T3SS tree was generated using FastTree v.2.1.10 based on our concatenated alignment [59]. All phylogenetic trees were visually enhanced using iTOL and branches with less than 50 % bootstrap support were collapsed [61].

### Population structure analysis

Population structure analysis was performed on the 58 sequenced representative *P. syringae* strains using structure (v2.3.4) [62] with the admixture model (PLOIDY=1, NUMINDS=58 and 20 000 Markov Chain Monte Carlo (MCMC) replications performed after 10 000 burn-in replicates). Genetic variants were generated using unitig-counter [63] (*k*-mer size=31), adapting the protocol outlined in the manual using in-house Python processing scripts to generate strain versus unitig pattern presence–absence tables as the input for structure. Unitig-counter was initially developed as an extension of *k*-mer based bacterial genome-wide association studies (GWAS); for a given genome sequence it implements a compressed de Brujn graph approach to identify and assemble overlapping *k*-mers into unitigs of varying sizes, which are then merged into unitig patterns if they have identical distributions across a set of genomes. Unitigs are ideally suited for population genetic analysis of bacterial populations with diverse pangenome content, where using traditional SNP-based approaches requiring a single reference genome can miss informative genetic variation.

Unitig patterns were generated to capture three levels of genomic content diversity: (i) all pangenome unitig patterns detected across the *Pseudomonas* pangenomes; (ii) unitig patterns associated with core-genome families; and (iii) unitig patterns associated with accessory-genome families. To do this, unitig patterns were identified separately for loci classified into core (100 % presence across *Pseudomonas* genomes) and accessory (<100 % presence across *Pseudomonas* genomes) gene families based on the pirate pangenome analysis, including only gene families containing no more than one locus per strain. In addition, for each set of unitig patterns, only those with maximum and minimum allele frequencies of 0.9 and 0.1, respectively, were chosen for structure analysis. This resulted in 46 951/50 207, 25 822/26 911 and 15 092/16 899 unitig patterns being kept for all pangenome, core and accessory genome subsets, respectively.

For each processed set of unitig patterns, structure was run iteratively on the Niagara Supercomputer Cluster over a range of maximum population values (MAXPOPS) from 2 to 10, with 15 replicates for each value. The number of variants selected (NUMLOCI) for each analysis was set to the number of unique unitig patterns identified above. The optimum number of populations/genetic clusters (*k*) was determined using the StructureSelector [64] webserver. Visualization of major and minor modes of the optimum *k* value were performed using the clumpak webserver [65] and were subsequently processed using in-house R scripts. Plots and phylogenetic trees generated by structure were visualized in R using the ggplot2, ggtree and ggstance R packages [66–69], with additional figure editing in Inkscape (https://inkscape.org).

### Identification and analysis of virulence genes

We specifically explored the distribution of three critical categories of *P. syringae* virulence factors in this study: T3SSs, T3SEs and phytotoxins. We analysed the distribution of different forms of T3SSs by first extracting all homologues, excluding those from the bacterial flagellin, for the following ten core T3SS structural genes from each genome using blast+ v0.2.6.0 (*E* value <1×10^−5^) [56, 57]: *hrcC*, *hrcJ*, *hrcN*, *hrcQ*, *hrcR*, *hrcS*, *hrcT*, *hrcU*, *hrcV* and *hrpV*. We then verified whether these structural genes were part of a T3SS island by characterizing their proximity to other T3SS structural genes. T3SS structural gene homologues that were part of a T3SS island were kept for further analysis. Based on our phylogenetic analysis of all T3SSs from the *Pseudomonas* genomes in our dataset, we identified six evolutionarily distinct versions of the T3SS: (a) the tripartite pathogenicity island (T-PAI), (b) the rhizobium-like pathogenicity island (R-PAI), (c) the single pathogenicity island (S-PAI), (d) the atypical pathogenicity island (A-PAI), (e) the fluorescens pathogenicity island (F-PAI), and (f) the corrugata pathogenicity island (C-PAI). Using a database containing a single representative structural gene suite for each T3SS (T-PAI, *P. syringae* PtoDC3000; R-PAI, *P. syringae* Pph1448a; S-PAI, *P. syringae* PchICMP3353; A-PAI, *P. syringae* PcoICMP19117; F-PAI, *P. fluorescens* PgeBBc6R8; C-PAI, *P. corrugata* PcoF113), we performed a blastp search (*E* value <1×10^−5^) where the proteome from each *Pseudomonas* genome in our dataset was queried against our T3SS library [56, 57]. If a hit was found, a T3SS structural gene was assigned to the version of the T3SS that the top hit was derived from. Finally, the presence of a given version of the T3SS was assigned to a strain if it contained more than half of the structural T3SS genes from a given version of the T3SS.

The distribution of 70 previously delimited T3SE families across strains was determined directly from our genome annotations, based on the T3SE database used during our annotation of each genome [34, 56, 57]. All effector genes were extracted from our genome annotations using the grep command based on a library keyword that was part of each of the 14 614 effector IDs in the library. These hits were then parsed into families based on the assignment of the most significant hit for each effector gene. The total number of effectors present in each genome represents the number of families present, where duplications of a given gene family within one genome are only counted once.

Finally, the distribution of eight known *Pseudomonas* phytotoxins or plant hormones that play critical roles in *P. syringae* virulence was assessed using a comparative approach. The phytotoxins analysed included coronatine, mangotoxin, phaseolotoxin, syringolin, syringomycin, syringopeptin and tabtoxin, as well as the plant hormone auxin. To assess whether functional toxins were produced by a given strain, we searched for the pathways required to synthesize each phytotoxin in each genome. Specifically, we performed a blastp search (*E* value <1×10^−5^; per cent identity >0.80), where representative query sequences involved in the synthesis of each phytotoxin were blasted against the proteomes of our 58 Turkey *Pseudomonas* strains [56]. Representative query sequences for each phytotoxin came from the following genomes: PtoDC3000 (coronatine), PsyBR2R (tabtoxin), PsyB728a (syringomycin), PsyUMAF0158 (phaseolotoxin, mangotoxin, syringolin, syringopeptin, auxin). A phytotoxin was considered present if more than half of the biosynthesis genes for the phytotoxin had significant hits in a given proteome.

## Results

### Samples

We collected and analysed a total of 175 *Pseudomonas* strains, including 169 strains from various Turkish provinces, and 6 strains from Germany (4), Holland (1) and Switzerland (1), which were included for comparative purposes (Table 1 and Dataset S1). The diseases caused by the 169 isolates from Turkey include: 39 citrus blast pathogens from lemon, mandarin orange and sweet orange; 38 bacterial speck pathogens from tomato; 33 knot disease pathogens from oleander, olive, fontanesia, myrtle and pomegranate; 20 pith necrosis pathogens from tomato; 10 bacterial canker pathogens from plum and apricot; 7 halo blight pathogens from bean; 6 bacterial blight pathogens from artichoke and pea; 6 leaf blight pathogens from muskmelon; 4 seedling blight pathogens from watermelon; 2 bacterial leaf spot pathogens from parsley; 2 leaf necrosis pathogens from muskmelon; and 2 uncategorized pathogens (Fig. S1). These isolates were collected from 1996 to 2018, but the majority of the collection was derived from recent outbreaks in the Adana, Hatay and Mersin provinces over the course of the last decade.

**Fig. 1.**
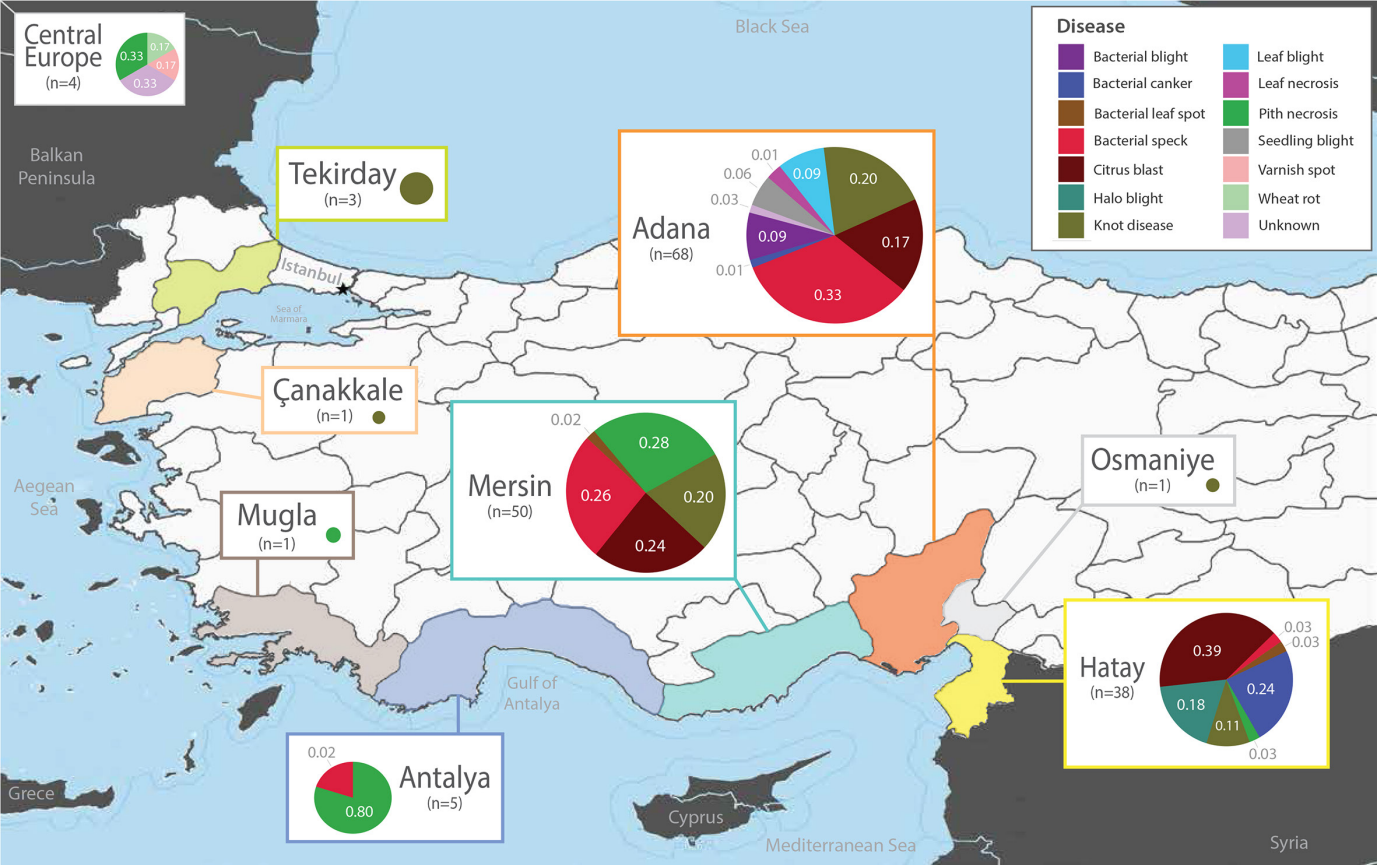
Sampling sites and disease metadata for the 169 *Pseudomonas* outbreak strains collected in Turkey between 1996 and 2018. Six additional *Pseudomonas* outbreak strains were also included from Germany (four), Holland (one) and Switzerland (one). Pie charts are proportional to the number of isolates collected at each site and illustrate the distribution of isolates that cause different diseases in the corresponding regions.

Among the broadly sampled diseases in this study (>10 samples per disease), we often find the same diseases dispersed across several geographical regions (Fig. 1; Dataset S1). For example, multiple outbreaks of citrus blast were sampled from 2012 to 2015 in the Adana, Hatay and Mersin regions, often appearing in multiple locations within the same year. Bacterial speck of tomato also appears to have dispersed to several provinces. After first appearing on tomato in the Hatay province in 1996, further bacterial speck samples were collected from Adana, Antalya and Mersin from 2012 to 2017. Similarly, knot disease isolates were sampled from multiple hosts across distant geographical ranges since its initial isolation in Canakkale in 2008. Finally, we collected tomato pith necrosis pathogens from Antalya, Mersin and Mugla in 2002 and 2003, and strains from a major outbreak in Mugla isolated in 2018.

### Emergence and dissemination of diverse disease-causing *Pseudomonas* strains in Turkey

**Fig. 2.**
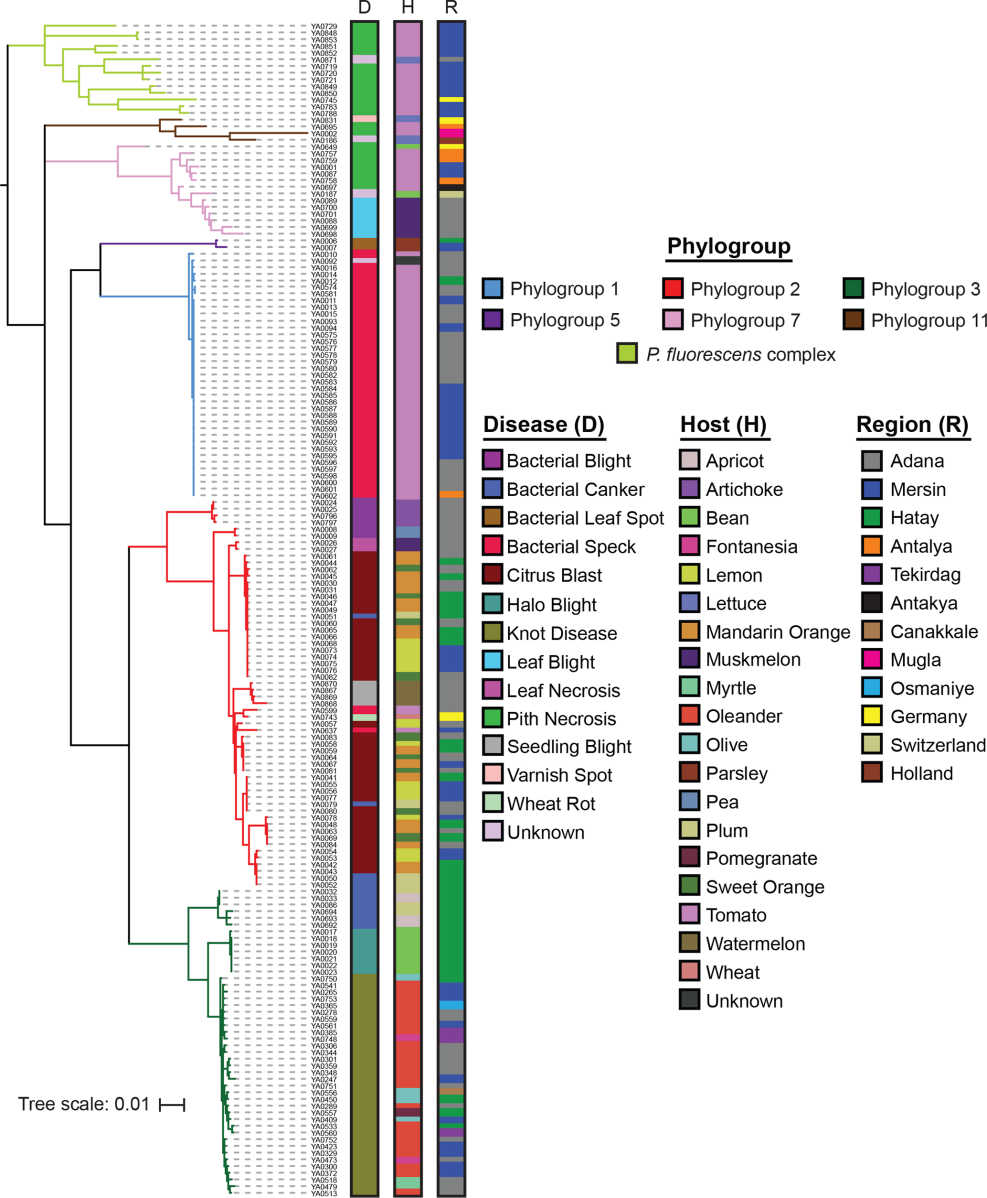
Evolutionary relationships between the 175 *Pseudomonas* strains isolated from diseased hosts in this study based on concatenated MLSA sequences of the *gapA*, *gltA*, *gyrB* and *rpoD* genes. Phylogroups were assigned based on the clustering of strains with representatives from 11 *P*. *syringae* phylogroups and 9 *P*. *fluorescens* phylogroups (Figs S2 and S3). The tree was rooted at the base of the *P. syringae* species complex and the tree scale reflects the number of stubstitutions per site. All alignments were generated with muscle and the tree was generated using FastTree, with an SH-Test branch support cut-off of 50 %.

We performed MLSA on the full collection of 169 Turkish strains and 6 comparative isolates using the *gapA*, *gltA*, *gyrB* and *rpoD* genes. Specific phylogroups were first assigned to strains using representative genomes from each established phylogroup in the *P. syringae* and *P. fluorescens* species complexes, with *P. aeruginosa* PAO1 as an outgroup (Fig. S2). This analysis confirms that the vast majority of our strains are part of the *P. syringae* species complex, particularly phylogroups 1, 2 and 3. However, a subset of the tomato pith pathogens clustered within the *P. fluorescens* species complex, and four strains (YA0848, YA0849, YA0850 and YA0853) appear to be outside of the phylogenetic boundaries of both *P. syringae* and *P. fluorescens*. While potentially interesting, some of these deep phylogenetic relationships may not be appropriately captured when using a small subset of loci. Therefore, these relationships require verification by more robust phylogenetic analyses using whole-genome data. Ultimately, we rooted the primary phylogenetic tree of 175 strains on the branch separating the *P. syringae* and *P. fluorescens* species complexes (Fig. 2).

The four diseases that were most broadly sampled in this study (citrus blast, bacterial speck of tomato, knot disease and tomato pith necrosis) were assigned as follows: *Citrus blast* (39) – all citrus blast pathogens cluster in phylogroup 2, although strains isolated from the same host do not cluster monophyletically, with lemon, mandarin orange and sweet orange isolates all being intermixed throughout the phylogroup. *Tomato bacterial speck* (38) – as we have observed previously [24], most tomato bacterial speck strains (36) are tightly clustered in phylogroup 1. However, we also identified two bacterial speck isolates in phylogroup 2. *Knot disease* (33) – all knot disease pathogens, commonly assigned to *P*. *savastanoi*, form a tight and distinct monophyletic clade in phylogroup 3 that is separate from the bacterial canker and halo blight strains in this phylogroup. As was the case with citrus blast, strains isolated from different hosts are largely intermixed within the knot pathogen clade. *Tomato pith necrosis* (20) – tomato pith necrosis pathogens make up our most diverse collection. All 12 strains from the 2018 outbreak in Mugla are part of the *P. fluorescens* complex, while the 2002 and 2003 isolates from Antalya, Mersin and Mugla cluster in secondary *P. syringae* phylogroups 7 and 11. This supports an independent origin of the 2018 pith necrosis outbreak from outside the *P. syringae* species complex.

Overall, the MLSA illustrates that while many isolates that cause the same diseases do cluster together phylogenetically, there are cases of independent, convergent evolution to the same host by highly divergent strains (tomato bacterial speck and pith necrosis). Furthermore, it is not uncommon to find the evolutionarily similar strains causing very similar disease phenotypes on multiple hosts, as is the case with citrus blast. This suggests that these strains share the ability to cause disease on a range of related host species, and that there has been only relatively minor divergence in the immune systems of these species. While we have less power to analyse smaller collections of bacterial canker of apricot and plum, bacterial blight of artichoke and pea, seedling blight of watermelon, leaf blight of muskmelon, leaf necrosis of muskmelon, halo blight of bean, and bacterial leaf spot of parsley, our results suggest that in most cases strains that cause these diseases are closely related. However, examples of convergent evolution are observed in our bacterial canker collection, where pathogenic lineages have arisen in both phylogroups 2 and 3.

We next performed whole-genome sequencing on 58 representative strains from the MLSA collection to obtain a higher-resolution picture of evolutionary relationships, including strains that cause: pith necrosis of tomato (18); citrus blast of lemon, mandarin orange and sweet orange (13); bacterial speck of tomato (8); bacterial canker of plum (5); bacterial blight of artichoke (4); seedling blight of watermelon (4); leaf blight of muskmelon (2); and leaf necrosis of muskmelon (2). Specifically, we focused on these strains because they represent either rapidly expanding outbreaks from multiple Turkish provinces (pith necrosis, citrus blast, bacterial speck) or are relatively new diseases that are not commonly isolated in Turkey. We analysed the evolutionary relationships between these strains using a pangenome analysis that also included the representative strains from each established phylogroup in the *P. syringae* and *P. fluorescens* species complexes, along with our *P. aeruginosa* PAO1 outgroup. A core-genome phylogenetic tree of all of these strains confirmed that phylogroup assignments in the *P. syringae* species complex from the MLSA were correct and allowed us to better resolve the more distant relationships of the *P. fluorescens* species complex (Fig. S3). All pith necrosis pathogens from the 2018 Mugla outbreak fall within the *P. fluorescens* species complex, with strains YA0783 and YA0788 clustering in the *P. corrugata* clade and the remaining 12 strains clustering in the *P. fluorescens* clade. Whole-genome sequencing also improved the within phylogroup resolution in the *P. syringae* species complex, allowing us to verify the distinct clades for smaller collections like leaf blight and leaf necrosis of muskmelon, while also showing that there are in fact distinct lineages of bacterial canker causing plum strains.

**Fig. 3.**
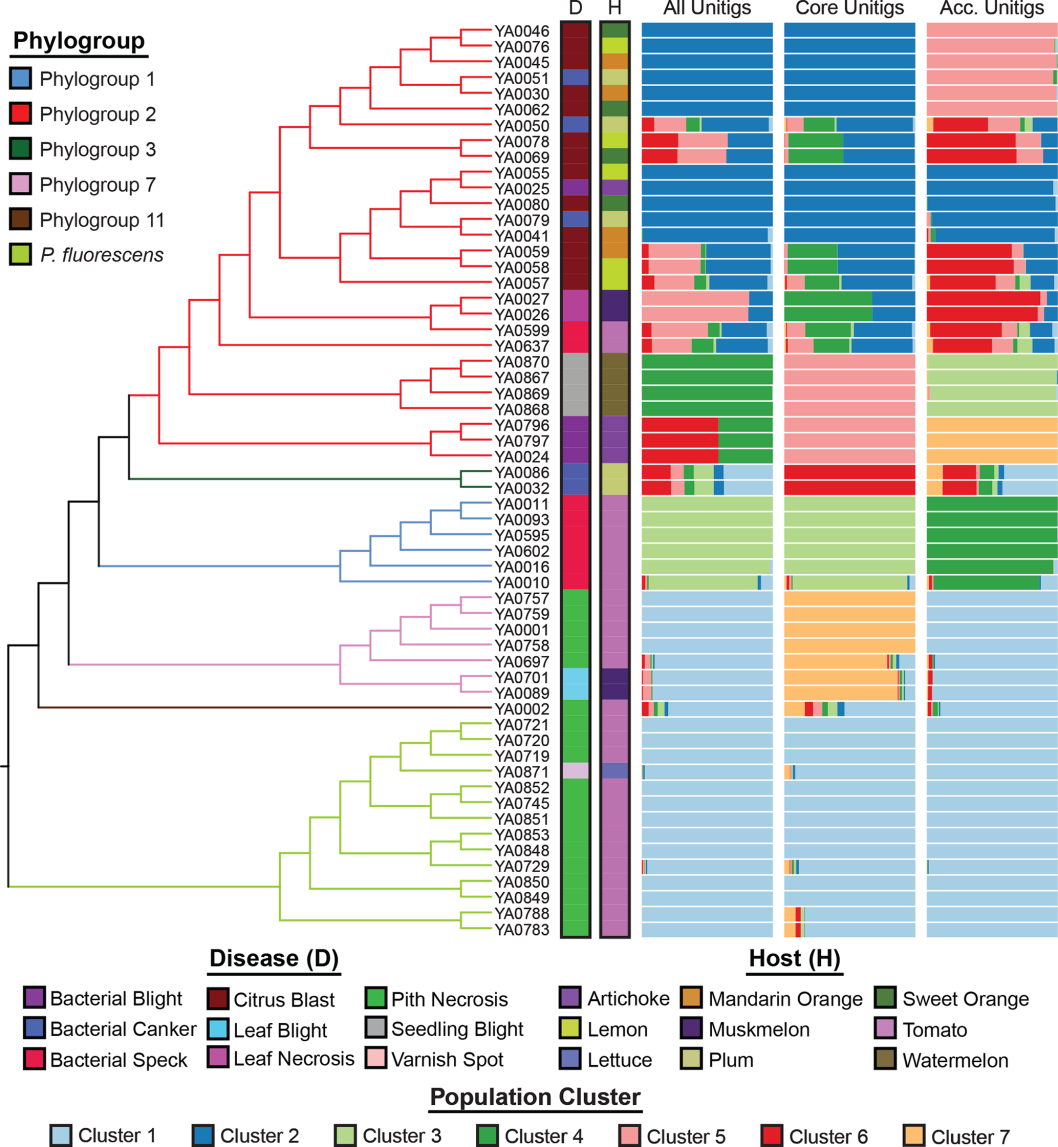
Population structure analysis conducted using structure v2.3.4 to assign all strains to population genetic clusters. For each analysis, the number of population clusters (*k*) was optimized using the Puechmaille method [93]. Bar plot panels indicate the clustering coefficients for replicate structure runs generated by clumpak [65] for three independent analyses comprising the unitig patterns of: the full pangenome, the core genome (100 % presence across strains) and the accessory genome (<100 % presence). The optimum *k* is six for the full pangenome, seven for the core genome and seven for the accessory genome. There is no relationship between the cluster colours of the three different structure analyses.

Finally, a gene content tree based on the presence–absence matrix of the entire pangenome reveals similar evolutionary relationships between strains, with all strains being assigned to the same phylogroups and independent clades distinguishing isolates that cause the same diseases (Fig. S4). A population structure analysis based on the pangenome, the core genome and the accessory genome was performed using unitig variants. In general, the results were consistent with the underlying phylogenetic relationships among strains (Fig. 3), but did provide additional resolution both within and between clades. Specifically, we find that tomato pith pathogens from the *P. syringae* and the *P. fluorescens* species complexes resolve into two distinct population clusters, based on core but not accessory unitig patterns. Among the phylogroup 2 strains, which comprise a substantial fraction of isolates sequenced, there exists a greater extent of population clustering, supported by both core and accessory genome unitig patterns. The accessory unitigs identified at least three population clusters with a degree of admixture among citrus blast (isolated 2012–2015), and tomato and muskmelon pathogens (isolated 2015–2017) (see Fig. 3 accessory unitigs panel – clusters 2, 5, 6), suggesting the possibility of a host jump facilitated by population differentiation. Collectively, the core-genome and pangenome data support the conclusion that while many isolates that cause the same diseases do cluster together phylogenetically and share similar gene content, the ability to cause the same disease has emerged independently in multiple lineages.

### Diversification of T3SS and T3SE repertoires

The T3SS is a critical virulence apparatus deployed by Gram-negative bacterial pathogens to directly inject an arsenal of T3SEs into the host cytoplasm. These effectors mediate the outcomes of host–pathogen interactions, because they can either promote virulence on susceptible hosts or activate an effector triggered immune response on resistant hosts [37, 70]. In order to explore the diversity of T3SSs and T3SEs across the genomes analysed in this study, we first extracted a set of ten T3SS core structural genes from each genome that are part of the *hrp*/*hrc* genomic island encoding the T3SS apparatus. We then created a concatenated alignment of all versions of the T3SS and built a phylogenetic tree to assess the evolutionary relationships between different T3SSs (Fig. S5).

**Fig. 4.**
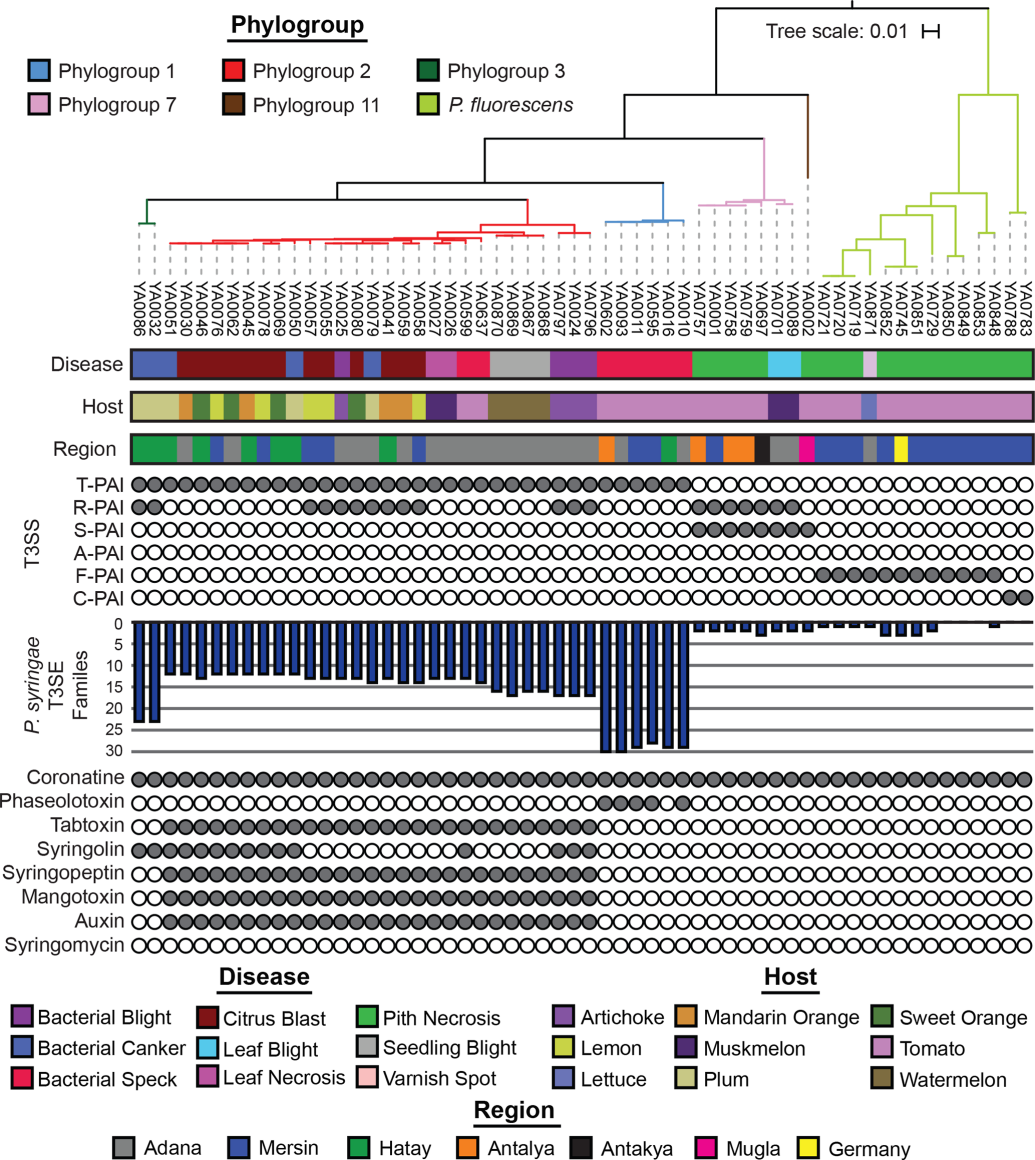
T3SS, T3SE and phytotoxin repertoires for each of the 58 representative *Pseudomonas* strains that were whole-genome sequenced. The phylogenetic tree was generated from a concatenated core-genome amino acid alignment using FastTree, with an SH-Test branch support cut-off of 50 %. The tree was rooted at the base of the *P. syringae* species complex and the tree scale reflects the number of stubstitutions per site. The six T3SSs analysed include the T-PAI from *P. syringae* PtoDC3000, the R-PAI from *P. syringae* Pph1448a, the S-PAI from *P. syringae* PchICMP3353, the A-PAI from *P. syringae* PcoICMP19117, the F-PAI from *P. fluorescens* PgeBBc6R8 and the C-PAI from *P. corrugata* PcoF113. The presence–absence of 70 established *P. syringae* T3SEs in each genome was used to quantify the collective effector repertoires in each strain. A phytotoxin was considered present if more than 50 % of the known protein sequences involved in the synthesis pathway had significant tblastn hits (1×10^−5^, >80 % identity) in the genome.

**Fig. 5.**
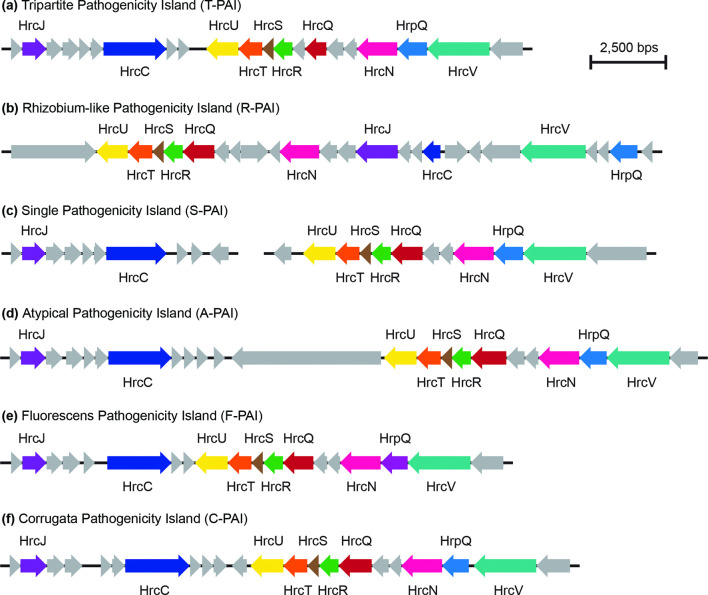
Genetic architecture of the T3SSs identified in this study. The genome architectures for each of the T3SSs was drawn from the following representative genomes: (a) T-PAI – *P. syringae* PtoDC3000; (**b**) R-PAI – *P. syringae* Pph1448a; (**c**) S-PAI – *P. syringae* PchICMP3353; (**d**) A-PAI – *P. syringae* PcoICMP19117; (**e**) F-PAI – *P. fluorescens* PgeBBc6R8; (**f**) C-PAI – *P. corrugata* PcoF113. All genes and non-coding regions are to scale.

Our T3SS tree reveals that five evolutionarily distinct versions of the T3SS are present in our 58 representative Turkey strains (Figs 4 and S5). While all phylogroup representative strains from the *P. syringae* species complex had at least one T3SS (Fig. S5), representative strains from the *P. chlororaphis*, *P. fragi*, *P. koreensis*, *P. mandelii* and *P. protegens* phylogroups in the *P. fluorescens* complex entirely lacked a *hrp*/*hrc* pathogenicity island, so they were excluded from the analysis. The canonical T-PAI T3SS is present in all *P. syringae* strains from phylogroups 1, 2 and 3, which is consistent with our prior observations that the T-PAI T3SS is conserved among strains from primary *P. syringae* phylogroups (1, 2, 3, 4, 5, 6 and 10) [24]. Alternatively, tomato pith and muskmelon leaf blight pathogens from secondary *P. syringae* phylogroups 7 and 11 harbour the single S-PAI T3SS. Pith necrosis strains from the *P. fluorescens* species complex each have one of two similar but distinct versions of T3SSs that we have termed the fluorescens (F-PAI) and corrugata (C-PAI) T3SSs, based on their sole presence in these *P. fluorescens* complex phylogroups. Finally, the *Rhizobium* R-PAI T3SS is a secondary T3SS that is present along with the T-PAI T3SS in a subset of *P. syringae* strains from phylogroups 2 and 3, and along with the S-PAI T3SS in phylogroup 7. Both the gene sequences (Fig. S5) and the genetic architecture (Fig. 5) of the R-PAI T3SS is quite different from the other forms of the T3SS, but its conservation in many genetic backgrounds already harbouring a T3SS suggests that it has an important and distinct function in these strains. Collectively, while T3SSs are clearly important virulence factors in *P. syringae* and *P. fluorescens* pathogens, we find that evolutionary history (i.e. phylogroup) is a much better predictor of the presence of a given T3SS than the disease caused by the strain.

**Fig. 6.**
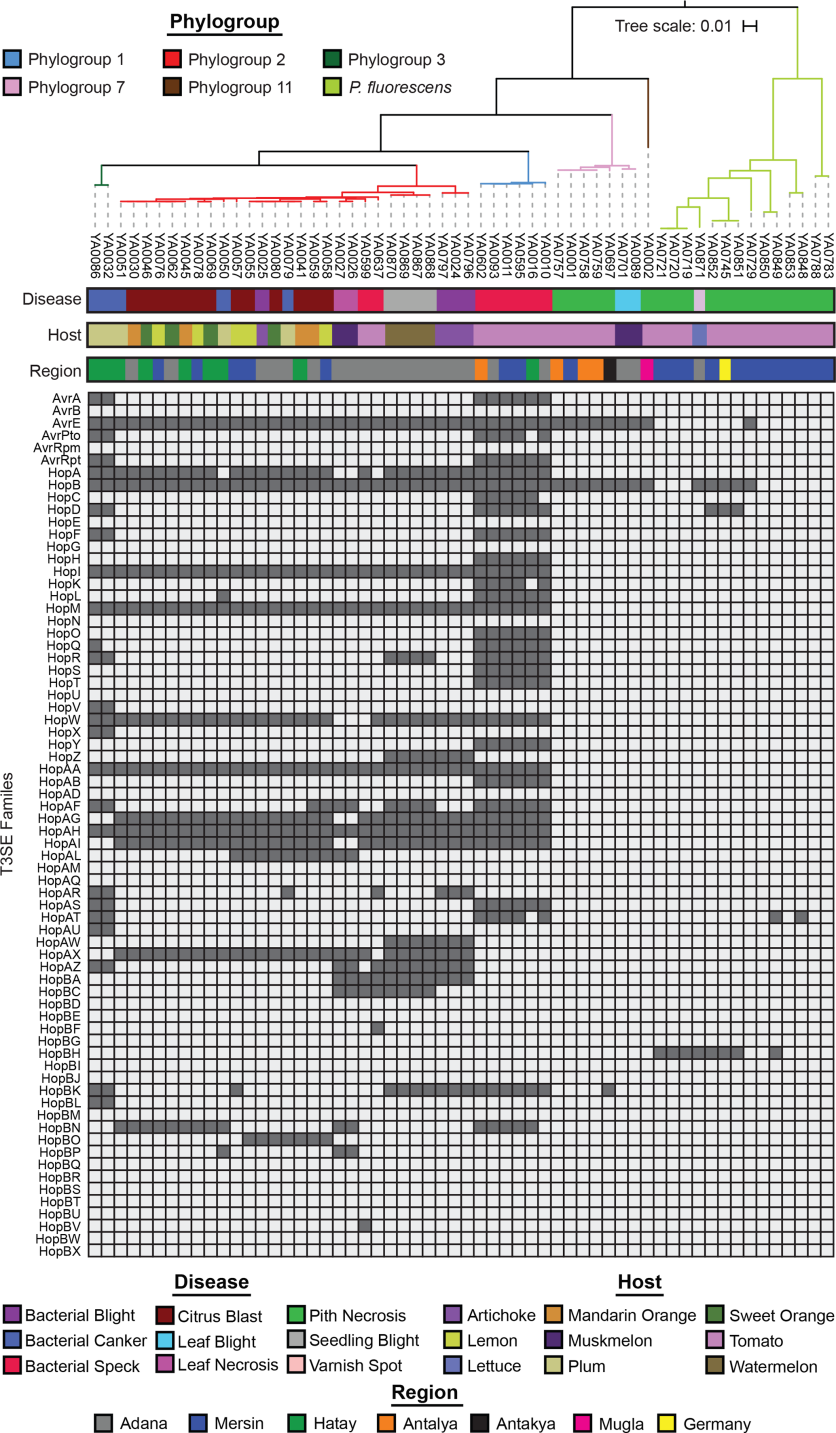
Complete T3SE repertoires for each of the 58 *Pseudomonas* strains that were whole-genome sequenced in this study. The phylogenetic tree was generated from a concatenated core-genome amino acid alignment using FastTree, with an SH-Test branch support cut-off of 50 %. The tree was rooted at the base of the *P. syringae* species complex and the tree scale reflects the number of stubstitutions per site. The 70 established *P. syringae* T3SE families that we delimited in an earlier study are listed on the left of the plot. A filled box indicates that at least one T3SE from the family is present in the strain and an empty box indicates that the T3SE is absent.

The presence–absence distributions of T3SEs across our representative Turkey strains also confirms our prior observations of the relationship between phylogroup and T3SE content (Figs 4 and 6) [34]. Primary phylogroup strains that harbour a canonical T-PAI T3SS all contain at least ten T3SEs, with phylogroup 1 strains harbouring the largest T3SE repertoires, phylogroup 3 strains harbouring T3SE repertoires of intermediate size and phylogroup 2 strains harbouring the smallest repertoires. Within phylogroups, we also see variation in T3SE repertoires (Fig. 6), with this variation segregating both within and between strains that cause disease on individual hosts. One notable example of T3SE loss from a specific disease lineage in phylogroup 2 is the loss of HopA, HopW, HopAG and HopAI in the muskmelon leaf necrosis lineage. These effectors are mostly conserved in the rest of the phylogroup. In contrast, HopBP is quite rare in phylogroup 2 and appears to have been acquired in this lineage. Similarly, watermelon seedling blight pathogens are the only strains in phylogroup 2 that harbour HopR, and lineages causing bacterial canker on plum display multiple lineage specific T3SE signatures. Secondary phylogroup strains causing pith necrosis on tomato and leaf blight on muskmelon all harbour fewer than three T3SEs, suggesting a reduced role of T3SEs in the virulence of these strains. Consistent with our prior observations [34], the AvrE and HopB effectors are the only conserved T3SEs in phylogroups 7 and 11 (Fig. 6). For their part, tomato pith pathogens from the *P. fluorescens* complex harbour more variable effector repertoires that include some combination of AvrE, HopB, HopD, HopAT and HopBH, although none of these effectors are universally conserved. Follow-up studies will be required to determine whether these effector signatures can explain the host specificity of epidemic *Pseudomonas* strains in Turkey.

### Distribution of *Pseudomonas* phytotoxins across strains

The phytotoxin profiles of this collection of plant pathogenic pseudomonads is also consistent with our earlier observations from a larger collection of sequenced *P. syringae* strains [24]. The ability to synthesize coronatine is conserved across all primary phylogroup strains of *P. syringae* (Fig. 4). Additionally, some phylogroup 1 strains that cause bacterial speck on tomato also synthesize phaseolotoxin, and the two phylogroup 3 strains that cause bacterial canker on plum also synthesize syringolin. Phylogroup 2 strains harbour the largest collection of phytotoxins and hormones, with the ability to synthesize tabtoxin, syringopeptin, mangotoxin and auxin also being conserved in this phylogroup. A subset of phylogroup 2 strains also synthesize syringolin. Finally, secondary *P. syringae* phylogroup strains and strains from the *P. fluorescens* complex appear to only be capable of synthesizing coronatine. These results are consistent with the idea that phytotoxins act as more general virulence factors across hosts, whereas T3SEs provide more host-specific adaptive benefits that cause them to undergo more frequent evolutionary turnover [24, 44]. The diverse phytotoxin and comparatively small effector contents observed in phylogroup 2 suggest that phytotoxin production may compensate for their reduced effector repertoires and that these strains may ultimately be better generalists because they are less likely to harbour immune eliciting effectors [44].

## Discussion

In this study, we analysed the population differentiation and virulence mechanisms of a diverse collection of *Pseudomonas* strains associated with recent agricultural outbreaks across Turkey. We found that common and widespread diseases on the same crop (e.g. tomato pith necrosis) can be caused by distinct lineages of *P. syringae*, while phenotypically similar diseases on distinct, but closely related, hosts (e.g. citrus blast on lemon, mandarin orange and sweet orange) can be caused by a single, closely related clade of pathogens. These observations raise interesting questions about the evolution of pathogen host specificity. For example, how frequently does convergent evolution onto the same host occur, and do these events typically entail minor variations of the same virulence mechanisms (e.g. functionally similar suites of T3SEs), or completely different virulence mechanisms? Do convergent virulence mechanisms target the same host immune complexes? Can we use these data to identify functionally similar groups of T3SEs or host immune complexes? Do all pathogen lineages have the potential to infect a broader array of hosts (i.e. are they host generalists) or is there variation with some lineages being more specialized while others are more generalists? Is host generalism driven more by specific pathogen virulence factors or common host immune factors? What role do putatively non-specific virulence factors, such as toxins, play in host generalism? Unfortunately, addressing these questions will require both broad (i.e. from many different hosts) and deep (i.e. many isolates from the same population) sampling, which is currently not available.

The convergence of disease symptoms among distinct evolutionary lineages highlights the adaptive flexibility of virulence mechanisms employed by *Pseudomonas* strains and is consistent with previous studies that have identified distinct lineages of isolates that cause similar disease symptoms on well-studied hosts like bean, tomato, cherry and kiwifruit [33, 71–73]. One possibility is that functional redundancy exists among key virulence factors like T3SEs and phytotoxins that has enabled these strains to cause similar disease symptoms despite their divergence [74–77]. This is more likely to be the case for bacterial speck and bacterial canker pathogens, which come from primary *P. syringae* phylogroups and, therefore, share a common ability to secrete T3SEs via the canonical T-PAI T3SS (Fig. 4). Furthermore, strains from primary phylogroups are also more likely to exchange virulence factors like T3SEs via horizontal gene transfer (HGT) [24], which may enable convergence to occur more readily. Indeed, a recent study of bacterial spot disease in the USA found that convergent acquisition of T3SEs in distinct pathogenic lineages through HGT resulted in hybrid emergence [78]. Other studies have found that categorically similar virulence arsenals (i.e. effectors and toxins) tend to turnover rapidly in different branches leading to the same diseases, though precise host-specificity-determining loci have remained elusive [33, 71, 72]. Our analyses of citrus blast, bacterial speck and bacterial canker are in line with the emerging consensus that while a complex genetic basis underlies host specificity, some shared features can be identified among strains that infect the same host, even when they occur in different clades. The repertoire for repertoire hypothesis [79], originally proposed in *Xanthomonas*, is likely also a good framework for understanding host specificity convergence in *P. syringae* because of the diversity and functional redundancy of the major virulence factors in this species complex.

Alternatively, molecular mechanisms independent of T3SE and phytotoxin repertoires may underlie the ability of divergent strains to cause the same disease. This seems more likely to be the case in tomato pith pathogens, which encompass a highly diverse collection of strains, do not harbour a canonical T-PAI T3SS, and secrete only a single phytotoxin. Interestingly, despite the fact that these strains do not harbour a canonical T-PAI T3SS, all of them do harbour either the S-PAI, F-PAI or C-PAI T3SS, which all have similar genetic architectures to the T-PAI T3SS. The presence of these T3SSs and the absence of homologues of the vast majority of *P. syringae* T3SEs raises the interesting possibility that these T3SSs may contribute to virulence by secreting other virulence genes not traditionally associated with *P. syringae* pathogens. Further studies will be required to determine whether these strains harbour currently unknown effectors. While some common host-associated genes have been identified in comparative genomic studies of host range, it is rarely the case that genetic knock-out or knock-in experiments fully explain host-specificity [71, 72], illustrating that we still have much to learn about the mechanisms underlying host compatibility. The diversity of these strains, particularly in the *P. fluorescens* complex, may enable the identification of these virulence factors via genome-wide association and predictive modelling analyses.

Our observation of overlapping disease-causing lineages that do not cluster by host also raises a number of key questions related to the generalism of different *P. syringae* strains. A common notion that has long been held in the *P. syringae* research community is that while the species complex as a whole has a broad host range, individual strains tend to be highly host-specific [1, 4, 71]. Recent work suggests that this notion is an oversimplification, as there appears to be considerable diversity in the host range of strains even within the same pathotype group [80, 81]. The citrus blast pathogens in this study were found in multiple clades of phylogroup 2, yet strains isolated from lemon, mandarin orange and sweet orange were intermixed across these clades. This observation is consistent with a diverse and overlapping continuum of host range that may be facilitated by frequent host jumps and explain why closely related strains that cause disease on different hosts are frequently observed [24, 80, 82]. Whether citrus blast pathogens from different clades have variable host ranges, as has been observed in some bean pathovars [71, 80], remains an open question that will be an interesting avenue for future work. Further pathogenicity assays that test the ability of our representative strains to cause disease across a panel of plants will allow us to characterize their host range and identify genomic features that drive generalism or specialism.

The emergence of recent outbreaks causing seedling blight of watermelon and leaf blight/necrosis of muskmelon in Turkey further suggest that naturally occurring host shifts are common. Both the watermelon seedling blight and muskmelon leaf necrosis pathogens arose in phylogroup 2, and were not accompanied by major changes to virulence repertoires. The propensity of phylogroup 2 strains to show weaker clustering by host of isolation may indicate that these strains have broader host ranges, which may be the result of their reduced reliance on potentially immune eliciting T3SEs for virulence [1, 24, 34, 42]. Because *P. syringae* is ubiquitous as an epiphyte in a wide range of agricultural and non-agricultural settings [3, 9–11, 83, 84], an increased use of phytotoxins and decreased reliance on T3SEs may facilitate more frequent host jumps.

The frequency of severe *Pseudomonas* outbreaks in Southern Turkey continues to threaten the agricultural output in these regions [17, 85–88]. However, it also provides us with an exceptional resource for studying the population genetics of disease in these diverse pathogens, which will ultimately help us to recognize outbreaks before they become widespread and engineer more broadly resistant crops. The strains collected in this study cause 13 different diseases on 19 different hosts, making it one of the most diverse single collections studied to date. Our analyses support a growing body of literature illustrating that host shifts are quite common and result in convergence of distantly related strains to the same host [33, 71–73, 81, 89–92]. We also find support for the notion that strains that have diversified to cause disease on multiple hosts do not form distinct monophyletic pathovars. This resource expands the growing collection of whole-genome data from plant pathogenic pseudomonads and will collectively enable critical insight into the population genetics of this globally significant pathosystem.

## Supplementary Data

Supplementary material 1

Supplementary material 2
