# Supplementary material for: Comparative genomic insights into the epidemiology and virulence of plant pathogenic pseudomonads from Turkey
Source: Microb Genom. 2021 Jul 6;7(7):000585. doi: 10.1099/mgen.0.000585 (PMC8477409; doi:10.1099/mgen.0.000585)
Supplement: Supplementary material 1 [file mgen-7-0585-s001.pdf]

Supplemental Material For

Comparative genomic insights into the epidemiology and virulence repertoires of plant pathogenic pseudomonads from Turkey.

Marcus M. Dillon, Tatiana Ruiz-Bedoya, Ceda Bundalovic-Torma, Kevin M. Guttman, Hae Kwak, Maggie A. Middleton, Pauline W. Wang, Sumer Horuz, Yesim Aysan, and David S. Guttman

Table S1: Primers used for Multi-Locus Sequence Analysis (MLSA) in this study.

Primer[1]	Sequence
gapA+264p *	CCGGCSGARCTGCCSTGG
gapA+312s	TCGARTGCACSGGBCTSTTCACC
gapA−874ps	GTGTGRTTGGCRTCGAARATCGA
gltA+174p	GCCTCBTGCGAGTCGAAGATCACC
gltA+513s	CCTGRTCGCCAAGATGCCGAC
gltA−1130s	CGAAGATCACGGTGAACATGCTGG
gltA−1192p	CTTGTAVGGRCYGGAGAGCATTTC
gyrB+271ps	TCBGCRGCVGARGTSATCATGAC
gyrB−1022ps	TTGTCYTTGGTCTGSGAGCTGAA
rpoD+147p	CAGGTGGAAGACATCATCCGCATG
rpoD+364s	GYGAAGGCGARATYGRAATCG
rpoD−1222ps	CCGATGTTGCCTTCCTGGATCAG

[1] PCR primers end in a “p”, while sequencing primer end in a “s”

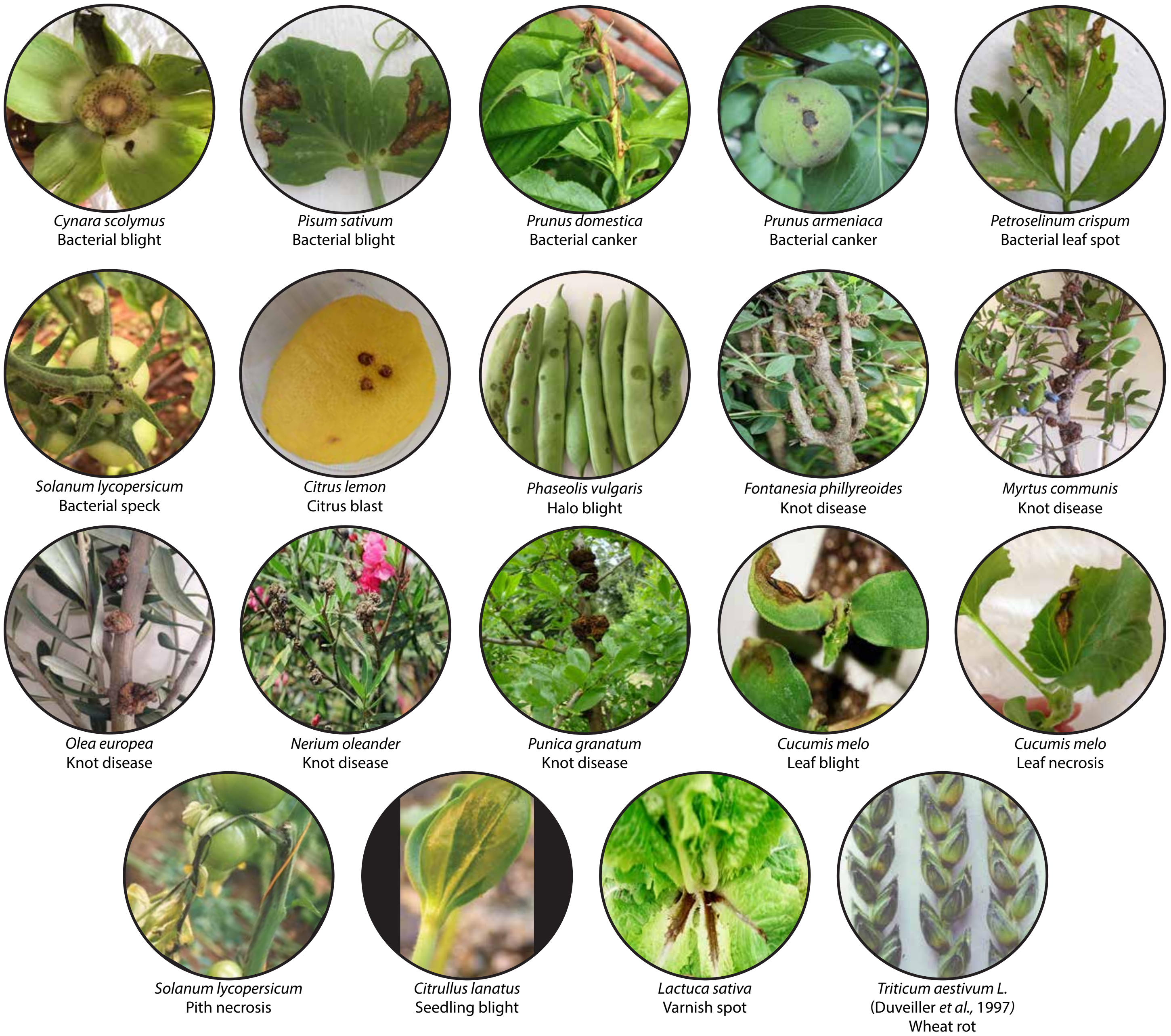

Figure S1: Images capturing the range of diseases caused by plant pathogenic pseudomonads isolated in this study, along with their associated hosts.

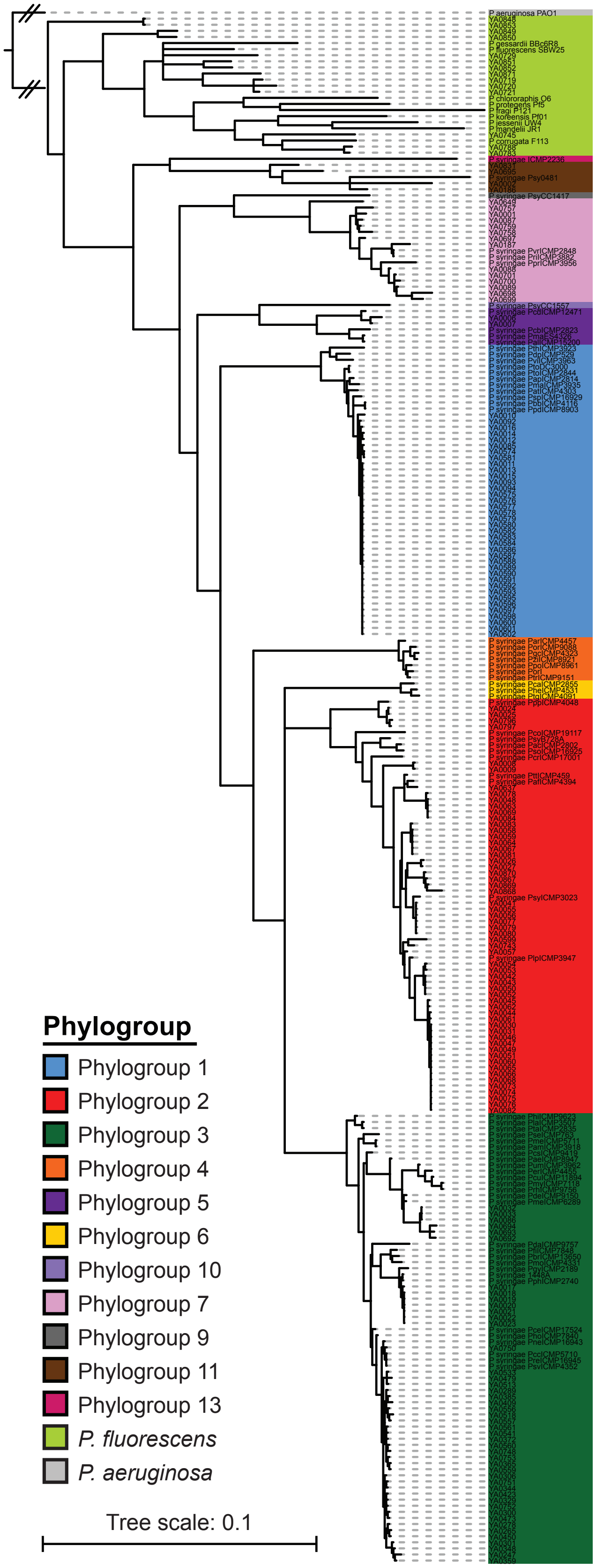

Figure S2: Phylogroup assignment of *Pseudomonas* strains based on multi-locus sequence analysis (MLSA). The phylogenetic tree was generated using a concatenated alignment of the *gapA*, *gltA*, *gyrB*, and *rpoD* genes. Representative strains from 11 *P. syringae* phylogroups and 9 *P. fluorescens* phylogroups (see Methods) were included in the analysis to assign species names phylogroups to the 175 strains analyzed in this study. All alignments were generated with Muscle and the tree was generated using FastTree, with an SH-TEST branch support cutoff of 50%. *P. aeruginosa* PAO1 was included as an outgroup to root the tree.

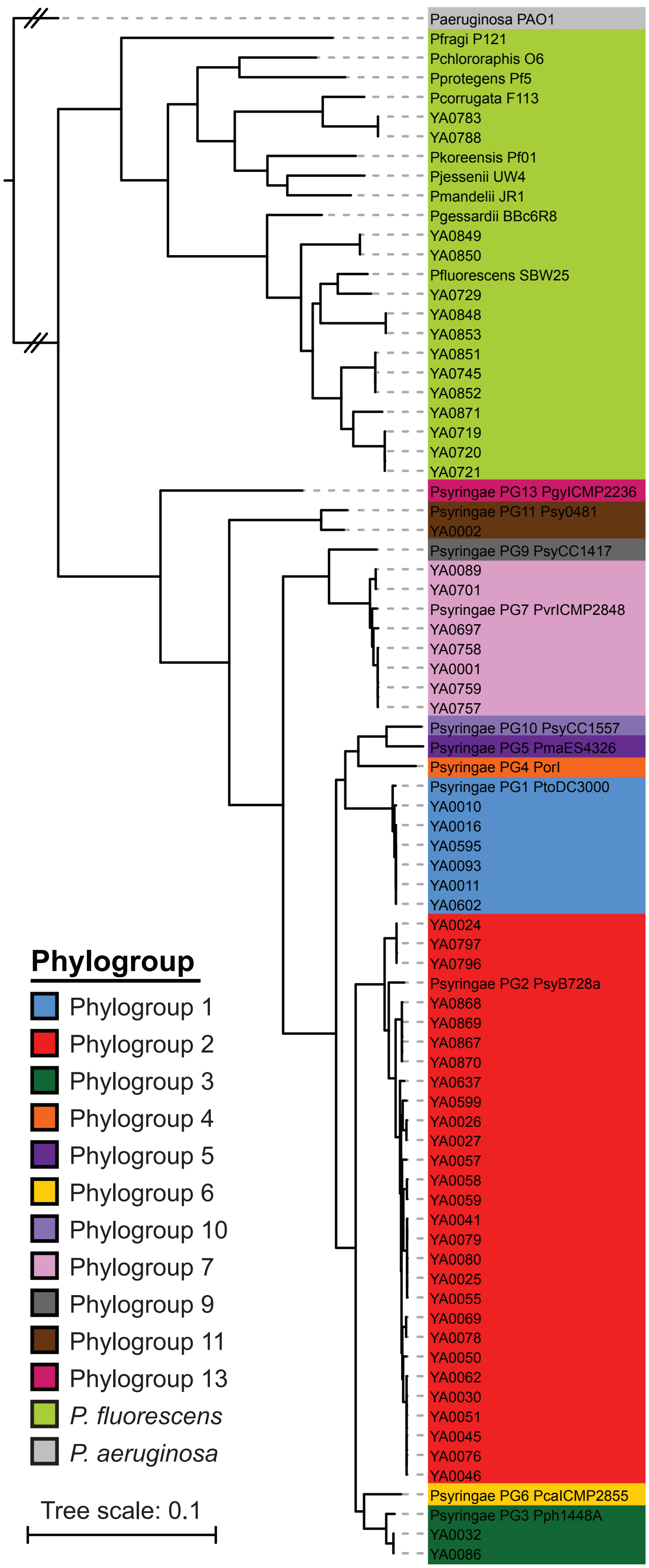

Figure S3: Phylogroup assignment of *Pseudomonas* strains based on a whole-genome sequencing. The phylogenetic tree was generated from a concatenated core-genome amino acid alignment using FastTree, with an SH-TEST branch support cutoff of 50%. Representative strains from 11 *P. syringae* phylogroups and 9 *P. fluorescens* phylogroups (see Methods) were included in the analysis to assign species names and phylogroups to the 58 representative strains whose genomes were sequenced in this study. *Pseudomonas aeruginosa* PAO1 was included as an outgroup to root the tree.

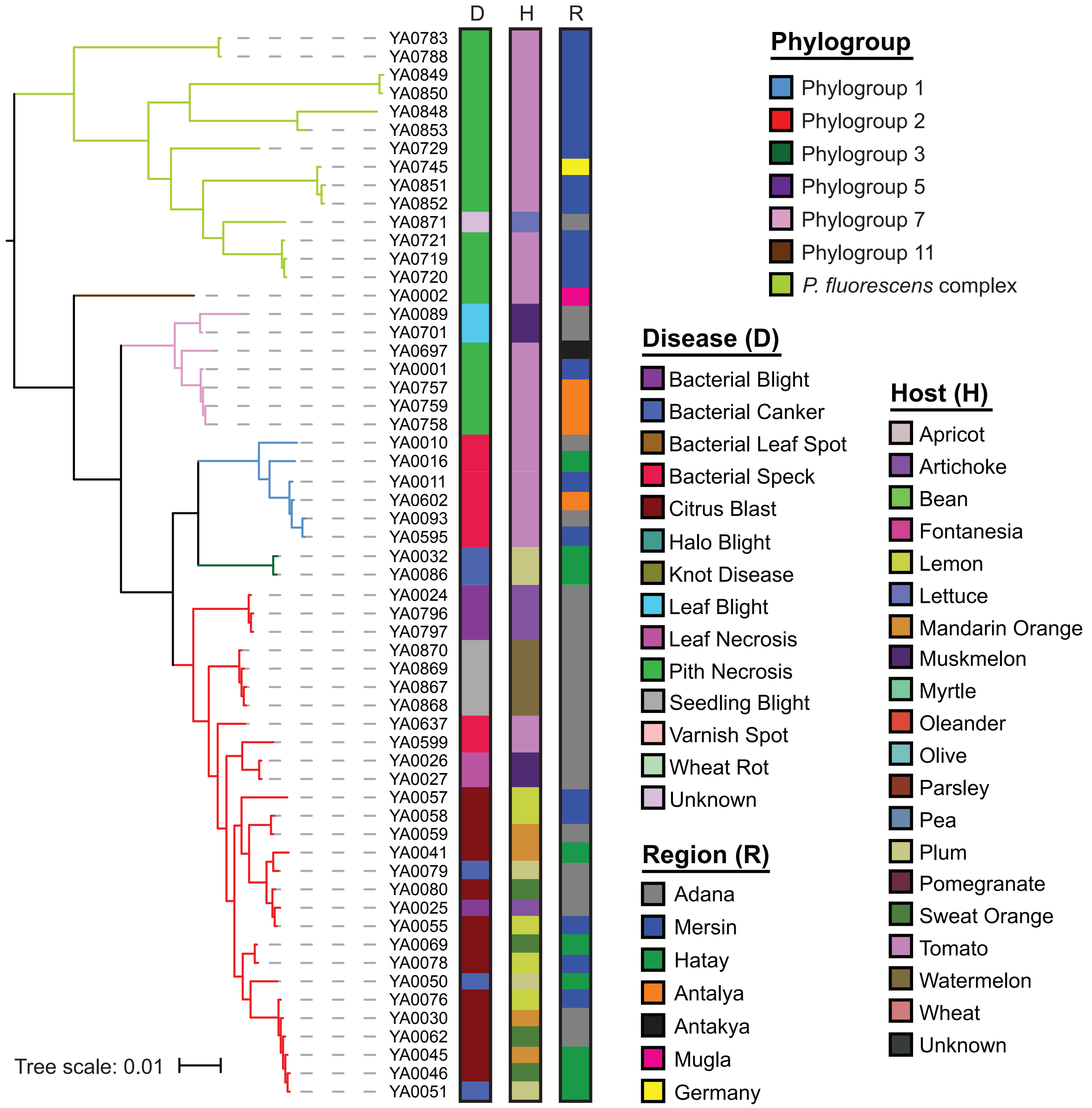

Figure S4: Evolutionary relationships between the 58 representative *Pseudomonas* strains sequenced in this study based on pan-genome content. The phylogenetic tree was generated using a presence-absence matrix that included all gene families present in our strain collection as input for FastTree, with an SH-TEST branch support cutoff of 50%. The tree was rooted at the base of the *P. syringae* species complex.

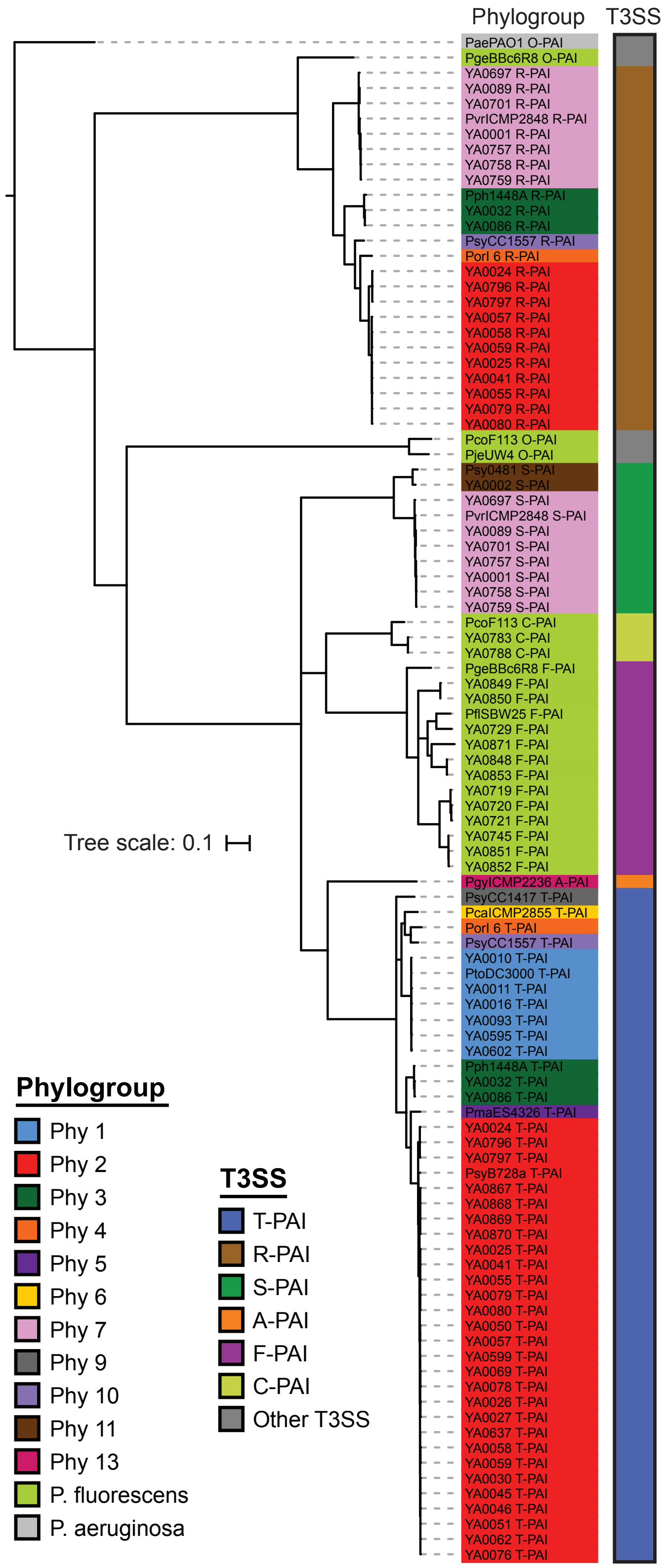

Figure S5: Evolutionary relationships between the type III secretion systems (T3SSs) identified in this study. The phylogenetic tree was generated using a concatenated alignment of the *hrcC*, *hrcJ*, *hrcN*, *hrcQ*, *hrcR*, *hrcS*, *hrcT*, *hrcU*, *hrcV*, and *hrpV* genes from each individual hrp/hrc pathogenicity island, including those from representative *Pseudomonas syringae* and *Pseudomonas fluorescens* complex strains from each phylogroup. Strains that have more than one T3SS appear multiple times on the tree. The T3SS from *Pseudomonas aeruginosa* PAO1 was used as an outgroup. All alignments were generated with Muscle and the tree was generated using FastTree, with an SH-TEST branch support cutoff of 50%.

Dataset S1: Complete metadata for all 175 *Pseudomonas* strains analyzed in this study.

SupplementalDatasets1and2.xlsx

Dataset S2: Genome assembly and annotation information for the 58 *Pseudomonas* strains that were whole genome sequenced and analyzed in this study.

SupplementalDatasets1and2.xlsx
